# Stable bulged G-quadruplexes in the human genome: identification, experimental validation and functionalization

**DOI:** 10.1093/nar/gkad252

**Published:** 2023-04-24

**Authors:** Csaba Papp, Vineeth T Mukundan, Piroon Jenjaroenpun, Fernaldo Richtia Winnerdy, Ghim Siong Ow, Anh Tuân Phan, Vladimir A Kuznetsov

**Affiliations:** Department of Urology, Department of Biochemistry and Molecular Biology, SUNY Upstate Medical University, Syracuse, NY 13210, USA; School of Physical and Mathematical Sciences, Nanyang Technological University, Singapore 637371, Singapore; Division of Bioinformatics and Data Management for Research, Research Group and Research Network Division, Research Department, Faculty of Medicine Siriraj Hospital, Mahidol University, Bangkok, Thailand; Bioinformatics Institute, A*STAR Biomedical Institutes, Singapore, Singapore; School of Physical and Mathematical Sciences, Nanyang Technological University, Singapore 637371, Singapore; Bioinformatics Institute, A*STAR Biomedical Institutes, Singapore, Singapore; School of Physical and Mathematical Sciences, Nanyang Technological University, Singapore 637371, Singapore; NTU Institute of Structural Biology, Nanyang Technological University, Singapore 636921, Singapore; Department of Urology, Department of Biochemistry and Molecular Biology, SUNY Upstate Medical University, Syracuse, NY 13210, USA; Bioinformatics Institute, A*STAR Biomedical Institutes, Singapore, Singapore

## Abstract

DNA sequence composition determines the topology and stability of G-quadruplexes (G4s). Bulged G-quadruplex structures (G4-Bs) are a subset of G4s characterized by 3D conformations with bulges. Current search algorithms fail to capture stable G4-B, making their genome-wide study infeasible. Here, we introduced a large family of computationally defined and experimentally verified potential G4-B forming sequences (pG4-BS). We found 478 263 pG4-BS regions that do not overlap ‘canonical’ G4-forming sequences in the human genome and are preferentially localized in transcription regulatory regions including R-loops and open chromatin. Over 90% of protein-coding genes contain pG4-BS in their promoter or gene body. We observed generally higher pG4-BS content in R-loops and their flanks, longer genes that are associated with brain tissue, immune and developmental processes. Also, the presence of pG4-BS on both template and non-template strands in promoters is associated with oncogenesis, cardiovascular disease and stemness. Our G4-BS models predicted G4-forming ability *in vitro* with 91.5% accuracy. Analysis of G4-seq and CUT&Tag data strongly supports the existence of G4-BS conformations genome-wide. We reconstructed a novel G4-B 3D structure located in the *E2F8* promoter. This study defines a large family of G4-like sequences, offering new insights into the essential biological functions and potential future therapeutic uses of G4-B.

## Graphical Abstract

**Graphical Abstract:**
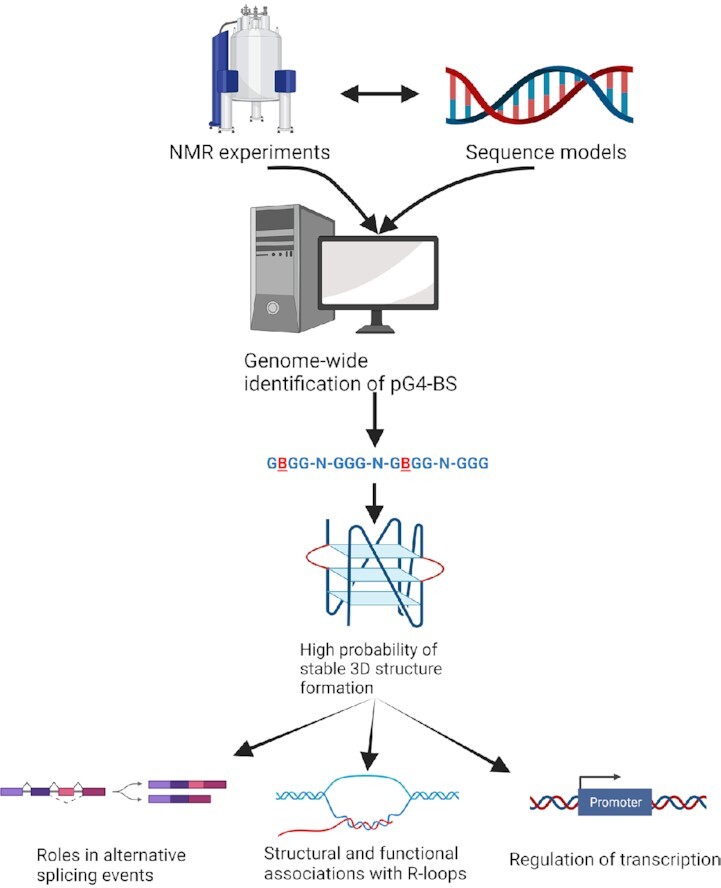
This study computationally identified and experimentally validated a large family of quadruplexes-like sequences, called stable bulged G4, offering new insights into the genome and gene functions and future therapeutic uses.

## INTRODUCTION

Nucleic acid sequences containing several continuous runs of guanines can form single-strand secondary structures called G-quadruplexes (G4s) (1,2). G4s are distinguished from the Watson–Crick double helix by the presence of stacked G-tetrads held together by Hoogsteen-type hydrogen bonding and further stabilized by monovalent cations (generally K^+^ or Na^+^) (3). Such structures in the genome could serve as key structural and functional modules involved in the regulation of gene expression (4,5), DNA replication and damage (6), the oxidative stress response (7,8), and local epigenetic modifications (9). Furthermore, G4 structures could serve as transcription factor (TF) binding sites, and specific DNA-binding proteins, thereby representing interesting cases where proteins bind to a specific DNA structure instead of a consensus sequence motif (10). G4s have also been implicated to have roles in the recruitment of CCCTC-binding factor (CTCF), a protein with critical functions in the organization of chromatin structure, gene silencing and transcriptional regulation (11,12). Due to their ubiquitous nature and involvement in a myriad of molecular processes, G4s play a key role as targets for proteins and other molecules involved in genome instability and are likely casual structural elements of genetic and other diseases (13–16).

The G4-forming DNA sequences (G4-S) themselves may also hold direct relevance to clinical practice, as G-rich genomic regions are prevalent in telomeric regions (17) and the promoter regions of oncogenes and tumor suppressors (18). For example, G4-S were identified in the promoters of oncogenes and tumor suppressors, such as *c-MYC* (19), *c-KIT* (20), *RET* (21), *hTERT* (22), *BCL2* (23,24) and *KRAS* (25).

G4 structures have become the object of intense study to define their potential as regulatory elements and/or therapeutic targets and, as a result, are increasingly considered as prospective drug targets (16). However, the sequences underlying G4 formation are diverse and, consequently, G4s are highly polymorphic (26). One of the main challenges in the development of G4-based drugs is the apparent lack of specificity to the targeted G4-S and G4s (27,28) and potential side effects. Despite this, promising results have been published about the application of G4 targeting drugs in melanoma cells (29,30), pancreatic cancer cells (31,32) and leukemia cells (33–35). The rRNA synthesis inhibitor compound CX-5461 is currently in Phase I clinical trials for the treatment of hematopoietic malignancies. Xu et al reported that CX-5461, and related compound CX-3543, bind to and stabilize G4 DNA structures *in vitro* and increase the numbers of G4s *in vivo* (36). Both CX-5461 and CX-3542 showed increased lethality in cancer cells with dysfunctional homologous recombination (HR) and non-homologous end-joining (NHEJ) DNA damage repair (DDR) pathways. BRCA1 and BRCA2 are required for proper HR and NHEJ to take place, rendering BRCA1/2 deficient tumors sensitive to treatment by both CX-5461 and CS-3542. These observations underline the idea that G4 binding ligands represent a potential treatment for HR/NHEJ deficient tumors.

Motivated by the clinical significance and the biotechnological perspectives of G4s, computational tools have been developed to locate potential G4-S in the genome of humans and other organisms (26,37). Different methodologies have been tried to identify such sequences (reviewed in (38)). For example, some newer G4 mining resources offer options for score-based filtering of identified G4-S to reduce false-positive results (39), or allow for the mining of conserved G4-S across different species (40,41). Traditionally, G4 mining algorithms utilize a regular expression that is based on experimental data describing G4 structure-sequence relationships. The general form of the regular expression used to identify canonical G4-S is **G_3+_N_1-7_G_3+_N_1-7_G_3+_N_1-7_G_3+_**, where subscripts indicate the number of nucleotides and N could be any combination of bases A, G, C and T. The essential feature of this sequence model is the presence of four clusters of three or more continuous guanines, which were thought to be a requirement for an intramolecular G4 formation. These guanine clusters are commonly referred to as G-stems, and the bases separating these stems are known as loops. Using the canonical regular expression, the total number of putative G4 structures in the human genome was reported to be around 350 000, with evidence pointing to G4 formation in many biologically relevant regions (37,42).

However, G4s can adopt a wide range of folding topologies based on the positioning of the four strands relative to each other, the DNA sequence and the length and orientation of the loops. Furthermore, the nucleotide composition and the lengths of G-stems and loops are key determinants of the intrinsic G4 forming ability of a given DNA sequence (39). The effects of loop length on G4 formation are still being researched, but evidence has shown that G4 structures with longer (>7 nucleotides) loops can exist (43). However, the consensus is that shorter loop lengths (≤4 nucleotides) confer higher thermodynamic stability over longer loops (44). In parallel to the broadening of the canonical G4-S definition, several types of *non-canonical G4s* have been reported over the years: duplex stem-loop containing G4s (45), G4s with truncated (26) or interrupted G-stems (46). We have studied the structure of a G4 containing a G-stem with a thymine insertion (47). Against the dominant notion, this sequence formed a G4 structure with all the guanines participating in the G-tetrad core and the thymine insertion(s) between the guanines forming a bulge in the G4-like structure. This was followed up by another study where the authors systematically investigated the possibility of the formation of bulges in an intramolecular G4 (46). All the G4-forming sequences tested were characterized by several non-continuous G-stems interrupted by other bases at different positions. We have reported on the first solution structure of intramolecular G4s formed by G4-S containing a single (or more) non-G nucleotide insertion(s) in G-stems, and we also have presented evidence that such non-guanine base ‘bulge’ containing sequences can fold into thermodynamically stable G4s with one or more bulges (G4-B) of diverse spatial variations. Recent works by our team members have further broadened the definition of potential G4-B forming sequences (pG4-BS) (48,49). Overall, these results provide *in vitro* experimental evidence that G4-S with a varying number, size, and type of bulges could exist and be capable of forming stable G4-B. Additionally, these findings underline that traditional sequence models used to identify putative G4-S likely underestimate the number of such structures. Several other groups have reported evidence that G4-B can form in the human genome under specific conditions (13) and that G4s with single nucleotide defects may be thermodynamically stable (26). Currently, the reconciliation of results acquired via G4-seq, G4-CUT&Tag and other NGS G4-like mapping methods, and computationally predicted data is still problematic, making the interpretation of biological studies complicated. In addition, it is difficult to identify the number and position of stable G4-BS using only experimentally derived datasets.

Potential canonical G4-S (pG4-CS) were observed to frequently co-localize with another type of non-B DNA structure, R-loops, which are also characterized by high GC-content. Transcriptional R-loop consists of guanine-rich RNA:DNA hybrid, which is thermodynamically more stable than the original DNA:DNA duplex and thus displaces the non-template DNA. R-loops are important regulators of gene expression and telomere lengthening, and many other physiological processes, but have also been implicated as sources of cellular and genotoxic stress, genome instability and diseases (50,51). Maizels *et al.* have shown evidence of the strand-specific co-localization of R-loops and pG4-CS at the genome level, hinting at overarching roles in the regulation and function of cancer-associated genes (1,15,52). They referred to these hybrid structures consisting of R-loops and G4s as G-loops.

Wongsurawat *et al.* (2011, Kuznetsov lab) has introduced quantitative models of the R-loop forming sequences (QmRLFS, http://r-loop.org/?pg=qmrlfs-finder), which provides highly accurate computationally predicted RNA:DNA hybrid/R-looping regions in natural or artificial DNA (53,54) and are used for high confidence mapping of the experimental ‘R-loop regions’ (54,55). QmRLFS is a non-template DNA that constitutes the R-loop initiation zone (RIZ), Linker and R-loop elongation zone (REZ) regions - motifs defined originally in class switch recombination regions of B-lymphocytes (56). RIZ and REZ of RLFS are highly enriched with repeated guanine clusters and pG4-CS genome-wide (http://r-loop.org/,^53^,^54^). A large proportion of genome-wide detected G4-like structures, as well as RNA:DNA hybrids, are overlapped with RIZ zones in a strand-specific manner and strongly co-localized/correlated in the vicinity of the transcription start site (TSS), 5′UTR, intron–exon junctions, splice sites, and transcribed enhancers (54). Structural and functional links between G4s and R-loops were observed in different contexts, such as the regulation of transcription (54,57), IgH class switch recombination (58), and the mechanism by which G4 binding factors elicit cancer cell killing activity (59,60). However, the degree of the structural and functional interplay between non-canonical G4 classes and R-loop formation, in particular pG4-BS and RLFS, has not been studied. In summary, due to the diversity of these non-canonical G4-like structures and the limitations of current experimental methods, identifying and defining their biological significance is challenging.

Here, we developed three novel G4-BS models (single-strand DNA sequence subsets) and utilize these models to identify pG4-BS in the human genome. We show that about 90% of human protein-coding genes contain at least one pG4-BS in either the promoter region or the gene body, that the high density of pG4-BS is preferentially localized in transcription checkpoints, including intron–exon junctions, around the transcription start site (TSS) and transcription termination site (TTS). Additionally, many of the pG4-BS overlap with other regulatory elements, such as DNase I hypersensitive sites and transcription factor binding sites (TFBS). Using gene ontology (GO) analysis we identify subsets of pG4-BS enriched in specific genes associated with GO terms related to transcriptional regulation, the nervous system, and diseases. We show that genes with the pG4-BS co-occurred on both chromosome strands and were enriched for GO terms over genes with single-strand pG4-BS occupancy. Co-localization analysis of experimentally detected G4s with our computational prediction data, including RLFS, reveals that the mapping resolution of current genome-wide experimental approaches targeting the identification of potential G4-S is not sensitive enough to fully capture the complexity and localization of the genome-wide frequency distributions (FD) of G4-like sequences. In addition, we consider pG4-BS on both template and non-template strands in promoters and study the associations of these complex back-forward G4 architectures with genes and pathways. The results of our analyses integrating our computational predictions with G4-seq and G4 CUT&Tag data strongly support the existence of G4-B conformations. Additionally, we use nuclear magnetic resonance (NMR) spectroscopy, circular dichroism (CD) spectroscopy and ultraviolet (UV) melting experiments for the identification of stable pG4-BS predicted by our models in cancer-associated genes. Finally, we provide the in-depth structural characterization of the G4-B formed by a novel pG4-BS located in the promoter region of the *E2F8* gene.

## MATERIALS AND METHODS

### Search algorithms

#### pG4-BS finding algorithm

In previous studies, we characterized the G4-forming ability of some artificial pG4-BS (46). Based on previous results on the stability of pG4-BS, we have developed a structural model and algorithm to identify potential pG4-BS genome-wide. According to our model, potential pG4-BS sequences could be identified using the following motif


$$\begin{equation*}G{I_x}G{I_x}G{\rm{\ }}{N_y}{\rm{\ }}G{I_x}G{I_x}G{\rm{\ }}{N_y}{\rm{\ }}G{I_x}G{I_x}G{\rm{\ }}{N_y}{\rm{\ }}G{I_x}G{I_x}G\end{equation*}$$

Here, ‘G’ denotes a guanine base, ‘I’ denotes any of the nucleotides A, T or C bases interrupting guanines with x number of nucleotides, ‘N’ denotes any bases (A, T, C or G) with y number of nucleotides. We have created seven sequence models for sequences that can potentially form G4-B (Supplementary Table S1). This classification is based on the number of intact G-stems and the overall number of bulged nucleotides. To search for potential pG4-BS in the human genome, we set the program parameters to $0\ \le x\ \le 3$ and $1\ \le y\ \le 3$, based on our previous study (47), and searched on both chromosome strands of the entire human genome sequence. We then eliminated canonical G4-S (*G_3+_N_1-7_G_3+_N_1-7_G_3+_N_1-7_G_3+_*) from our sequences as well as pG4-BS sharing at least one nucleotide on the same strand with any pG4-CS. In the pG4-BS set, we kept only sequences with at least two G-tracts without bulge(s) and a maximum of two bulges in the sequence. In addition, we did not allow more than a single G linker between G-stems and excluded pG4-BS with continuous cytosine repeats (e.g. CC, CCC, CCCC).

#### pG4-CS finding algorithm

While tools exist that predict G4-S (38), traditional methods (i.e. Quadparser (37)) do not discriminate individual multiple G4-forming sites from a single region of DNA. Therefore, we have developed a computer algorithm in Python to identify individual G4-CS. We have set the search rule (G_3+_N_1-7_G_3+_N_1-7_G_3+_N_1-7_G_3+_; where N refers to any base) depending on the parameters traditionally used by other search algorithms. We used our program to identify individual G4-S in both strands of the human genome (Hg38).

#### Repeat-element exclusion

In the case of both pG4-CS and pG4-BS datasets, we identified and removed the sequences that coincide with low complexity regions or repeat elements, such as SINE, LINE, tRNAs or snRNAs. The full dataset of repeat elements used in our analysis, as well as the full list of repeat elements considered, is available from the UCSC Genome Browser RepeatMasker track (https://genome.ucsc.edu/cgi-bin/hgTrackUi?g=rmsk).

### DNA sample preparation

Unlabeled and site-specific labeled DNA oligonucleotides were chemically synthesized on an ABI 394 DNA/RNA synthesizer. The oligonucleotides were de-protected, purified, dialyzed successively against ∼20mM KCl and water, and prepared in a buffer containing 20 mM KCl and 20 mM potassium phosphate (pH 7.0).

### NMR spectroscopy

Nuclear magnetic resonance (NMR) experiments were performed on Bruker 600 and 700 MHz spectrometers at 25°C, unless otherwise specified. Resonances for guanine residues were assigned unambiguously by using site-specific low-enrichment 15N-labelling (61) and site-specific 2H labeling (62). Spectral assignments were assisted by NOESY, COSY, TOCSY and 13C-1HHSQC, as previously described (63,64). All spectral analyses were performed using the program FELIX (Felix NMR, Inc.).

### CD spectroscopy

Circular dichroism (CD) spectra were recorded on a JASCO-815 spectropolarimeter over the range of 220–320 nm using a 1-cm path length quartz cuvette with a reaction volume of 500 μl. For CD-melting experiments, cooling and heating were successively performed across the temperature range of 15–95°C over a total of 14 h. At intervals of 1°C, the full spectrum was recorded as an average of three scans, the spectrum of the buffer was subtracted, and the data were zero-corrected at 320 nm. The molar ellipticity at 295 nm was extracted for melting analysis. Two baselines corresponding to the completely folded (low temperatures) and completely unfolded (high temperatures) states were manually drawn to determine the fractions of folded and unfolded species during the melting process.

### UV melting experiments

The stability of G-quadruplexes was characterized in UV melting experiments conducted on a JASCO V-650 spectrophotometer. Experiments were performed with 1-cm path-length quartz cuvettes. DNA concentration ranged from 4 to 6 μM. Solution contained 30 mM KCl and 20 mM potassium phosphate (pH 7.0) or 6 mM KCl and 4 mM potassium phosphate (pH 7.0). Samples were initially incubated at 90°C for 10 min and then cooled down to 20°C at a rate of 0.2°C/min; after a delay of 10 min, they were heated back to 90°C at the same rate of 0.2°C/min. Absorbance at 295 nm was recorded as a function of temperature ranging from 20 to 90°C. The linear pre- and post-transition regions of each absorbance-versus temperature curve were taken as the baselines corresponding to the completely folded (low temperature) and completely unfolded (high temperature) states. The fractions of the folded and unfolded states were derived by taking the ratio of the differences between the baselines and the experimental curve at each temperature.

### G4-seq data

To evaluate the enrichment of our computational pG4-BS/pG4-CS data with G4-seq signals, we downloaded available G4-seq data from GSE110582 (65). During the improved G4-seq method, each read is sequenced twice: first, under non-G4 stabilizing conditions (e.g. Li^+^ buffer), and second, under G4-stabilizing conditions (e.g. K^+^ or K^+^ and pyridostatin (PDS) containing buffer). Following sequencing, the results of the first and second run are compared and based on the detected mismatches (that are due to folded G4 structures interfering with the sequencing process) the potential sites of G4 formation (also known as G4-seq hit scoring regions) are mapped and quantified. From the downloaded datasets, using the preprocessing and peak calling methods reported in (65), we extracted the sites of potential G4 formation and combined them into a single dataset. This data contains ‘pooled’ potential G4-forming sequences detected either under milder (K^+^ buffer) or stronger (K^+^ and PDS buffer) G4-stabilizing conditions, thereby capturing the highest number of G4-seq identified regions (*n* = 949 162). The selected G4-seq hit scoring regions may or may not contain (or overlap) with computationally predicted G4-like sequences. We also specified a subset of G4-seq hit scoring regions that were observed under both G4-stabilizing conditions (both K^+^ and K^+^ + pyridostatin (PDS)). We termed this subset of G4-seq signals, ‘higher confidence’ G4-seq regions (*n* = 200 582).

### G4 CUT&tag data

Here, we reproduced the computational pipeline reported in the Supplementary Material of Li *et al.* (5). We downloaded the raw data from the SRA database for the G4 CUT&Tag samples (Supplementary Table S7.1). We used Bowtie2 v2.4.5 to align reads to the human genome (UCSC hg38) with the parameters ‘–no-unal’ and ‘–sensitive’. We only included uniquely mapping reads in the analysis using the *markdup* function of SamTools v1.6.1. We performed peak calling on DNA input controls from NCBI (SRX5466670, SRX3358201, SRX5449793, SRX6858029 and ERX4517391) and MACS2 v2.2.7 with default parameters and a *q*-value cutoff of 1.00E-12. To compare data with G4-S models and G4-seq peak regions, we merged all peaks from all cell lines. In total, we identified 45968 merged peak calling regions in the DNA samples of cancer cell lines K562, HeLa, LM2 (MBD-231-LM2), SW1271, and two replicates of the embryonic kidney HEK293T cell line.

### Enrichment analysis of pG4-S (pG4-BS/pG4-CS) in G4-seq and G4-CUT&tag peak regions

In order to count pG4-S enrichment in experimentally detected G4 signal regions genome-wide, we use a computational method that identifies a set of ‘background sequences’. In such a set, the number of sequences is the same as in the representative pG4-S set. In addition, the number of randomly chosen sequences follows the same FD of sequence lengths as in the representative pG4-S set, and random sequences that overlap with the original location of pG4-S are excluded.

In particular, let's examine the enrichment scoring method for merged G4-S (merged pG4-BS and merged pG4-CS) in G4-seq peak regions. First, the algorithm selects random samples of 10 000 sequences from our computationally predicted merged pG4-S dataset. Next, it selects 10 000 random background sequences from the human genome, controlling for length and selecting only sequences that do not overlap with the original position of any pG4-S. After that, the algorithm counts how many background sequences overlap with at least one G4-seq region. Positive and negative categorical events are the overlapped background sequences with the G4-seq peak region versus the non-overlapped background sequences. If at least one nucleotide is shared between two sequences of different sets, the event is positive. Using the positive and negative event numbers for pG4-S and background sequences relative to G4-seq peak regions, our method constructs 2 × 2 contingency tables and calculates the enrichment score (ES). ES is defined as the ratio between the proportion of positive G4-seq events in the set of the G4-S and the ratio of positive G4-seq events in the background sequence set. For statistical testing, and calculation of the Standardized Difference, 2-sided *P*-value, and confidence intervals, we used the Ratio of Two Binomial Proportions test in Cytel Studio 9. This method is also applied to calculate ES in the case of G4-CUT&tag datasets.

The few or no replicates, low signal region resolution and technical biases in available NGS-based G4 mapping data limit their use as a reference for identifying and validating pG4-BS and pG4-CS (see below). However, the quality of such datasets could be improved in the context of identifying and selecting stable pG4-S (pG4-CS/pG4-CS)-positive data from G4-seq/CUT&Tag peak regions. In these cases, our enrichment analysis can be used to filter out technically problematic and false positive experimentally defined signal peak regions by counting pG4-S(pG4-CS/pG4-CS)-positive G4-seq (or G4-CUT&tag) signal peak regions and pG4-S(pG4-CS/pG4-CS)-positive background signal peak regions. These cases were handled using our method.

## RESULTS

### Structural study of pG4-BS reveals sequence constraints required for G4-B formation

Bulges are structural elements of G4s, which form protrusions of bases from the G-tetrad core. Bulges occur due to the presence of non-guanine bases in one or more G-tracts of the G4 forming sequences. Previously, we have shown that G4 structures could incorporate bulges (46) or other non-canonical G cluster-rich short DNA sequences, such as duplex stem-loops (45). We and other groups have also reported on the potential incorporation of bulges and their effects on G4 stability (26,48,49,66). The ability of such sequences to form thermodynamically stable structures genome-wide may be due to the existence of diverse classes of non-canonical G4-like structures, for instance, distinct subsets (motifs) of G4-B (26,46,47). However, the specific sequence characteristics that would allow for the identification of G4-B sequences are unknown.

**Figure 1.**
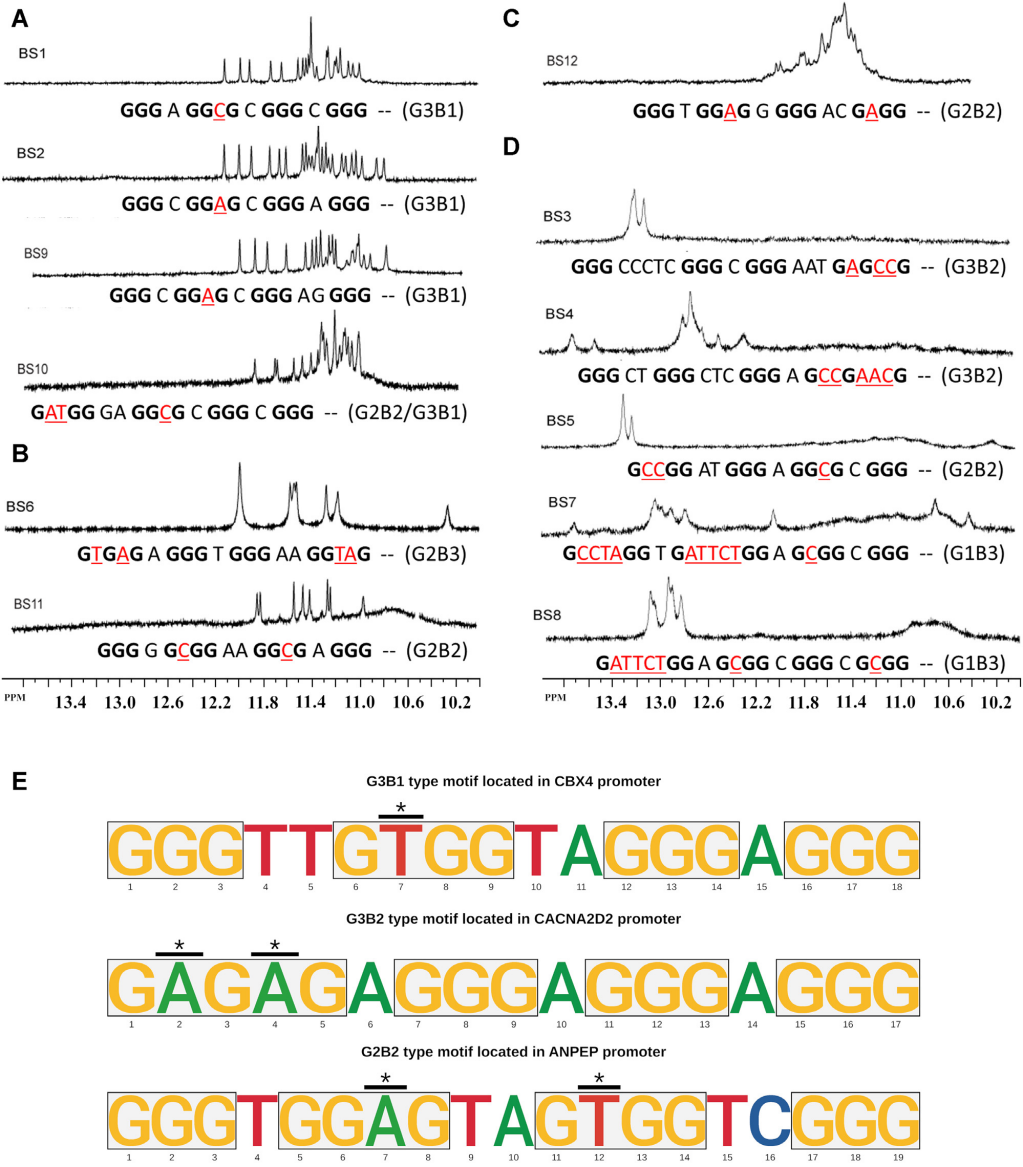
(**A**) NMR spectra of sequences with one single nucleotide bulges indicate the presence of a single stable G4 conformation. (**B, C**) NMR spectra of sequences with two bulges located in either the same or the different G-stems suggest the formation of G4s that lack a single stable conformation. (**D**) The incorporation of more than two bulges, excessive bulge length and higher cytosine content abolish G4-formation in the tested sequences. (**E**) Sequence logos of three pG4-BS sequences identified in the promoters of human protein coding genes, each belonging to one of the three sequence models. Nucleotides are color-coded: Guanine with yellow, Thymine with red, Adenine with green and Cytosine with blue. The guanine clusters in each sequence are highlighted with yellow-colored rectangles. The black line with the asterisk above a given nucleotide indicates that it is interrupting a G-stem and would be excluded from the 3D G4 structure in the form of a bulge.

In this study, we constructed several *in vitro* sequence models that varied based on the number of insertion sites and the number of uninterrupted stems (Supplementary Table S1). We aimed to select the models corresponding to sequences with a high probability of G4-B formation and use them to identify the locations of such sequences genome-wide. To specify our G4-like models and select the most appropriate nucleotide sequence patterns, we started with a conformation stability analysis of 12 representative model sequences from our initial search (Figure 1A–D). First, we focused on pG4-S with three guanines in each G-stem and short (≤3) loops. All tested sequences contain 12–13 guanines that could theoretically form a three-layered G4-B. These pG4-BS contain non-G (C, T or A) interruptions in up to three of the four stems. Additionally, the tested sequences are diverse regarding the length of their loops and bulges (see Materials and Methods).

Our results showed that sequences with bulges of length one could form three-layered G4s (Figure 1A). We also observed that G4 formation by the sequences that contain two bulges, albeit their NMR spectra suggest that these are less stable (Figure 1B, C). In our experiments, some sequences with longer loops (>3 bases), more than two interrupted stems, or contiguous cytosine bases (e.g. CC, CCC) content failed to form G4s (Figure 1D). Also, our results agree with our previous findings that G4s with a single bulge can be more stable than G4s with more bulges (46). Furthermore, our results indicate that longer loops and contiguous cytosine bases, and enrichment of the cytosines decrease the thermodynamic stability of G4-B.

### *In silico* identification of potential bulge containing G4 quadruplex sequences (pG4-BS) in the human genome

**Figure 2.**
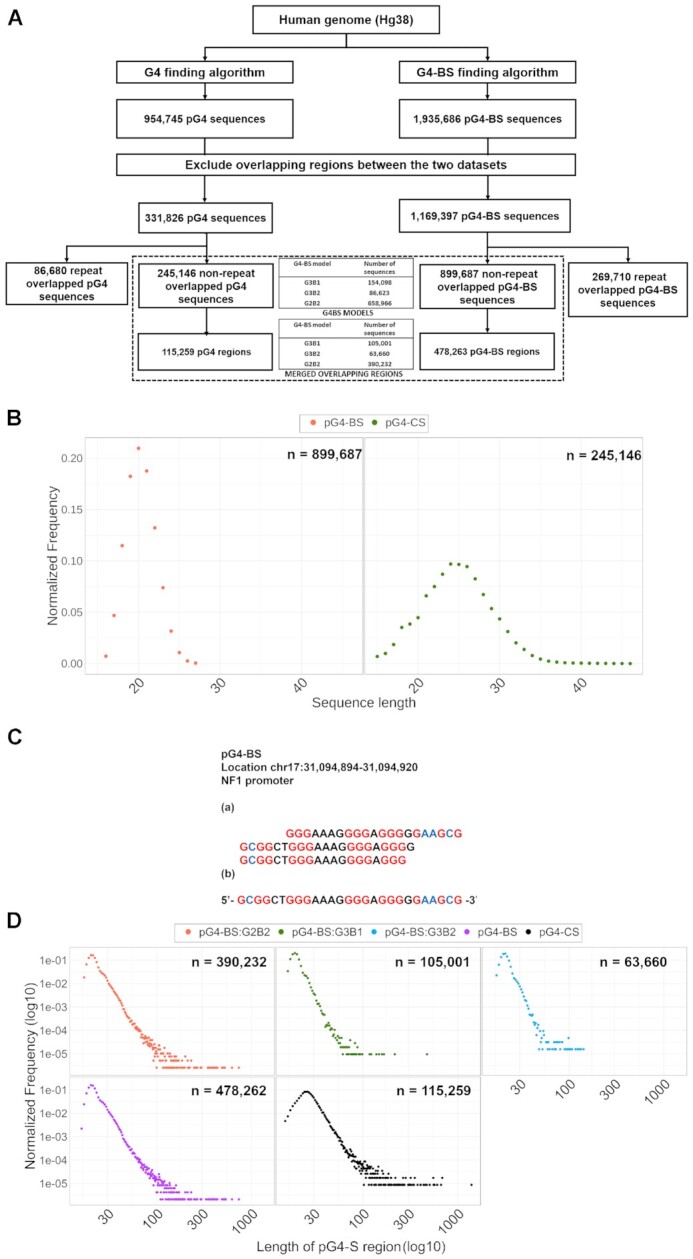
(**A**) Flow chart of the search algorithms used for the identification of pG4-BS and pG4-CS in the human genome. Overlapping sequences between pG4-CS and pG4-BS datasets were excluded, as well as sequences that overlap with any repeat elements. Finally, sequences were merged into pG4-CS regions and pG4-BS regions. (**B**) FD functions of the lengths of unmerged pG4-BS (mean: 20.3, median: 20, mode: 20, standard deviation:1.83) and pG4-CS (mean: 24.8, median: 25, mode:24, standard deviation: 4.14). (**C**) Example of multiple pG4-BS sharing G-stems. As we cannot reliable tell which one of the overlapping sequences partake in G4 formation, we merged overlapping pG4-BS and pG4-CS sequences into a single pG4-BS region or pG4-CS regions, respectively. (**D**) FD functions of the lengths of merged regions of three G4-BS models: G2B2 (mean: 22.7, median: 21, mode: 20, standard deviation: 6.46), G3B1 (mean: 20.02, median: 19, mode: 19, standard deviation: 3.74), G3B2 (mean: 21.6, median: 21, mode: 21, standard deviation: 3.53), all pG4-BS (mean: 22.79, median: 21, mode: 20, standard deviation: 6.48), and pG4-CS (mean: 27.75, median: 26, mode: 25, standard deviation: 11.95). The numbers following ‘n’ in the upper right corner of plots indicate the total number of merged regions in each of the categories. The frequency of individual pG4-S regions was counted for each given length and normalized for the total number of pG4-S in each of the considered categories. For bulk pG4-BS and pG4-CS, the power law-like properties of the tails of the distribution functions are illustrated by the fitted red and brown lines, respectively.

Here we sought to identify potential sequences capable of forming G4-B (pG4-BS) genome-wide. Based on our previous results, we selected three G4-BS models that correspond to the tested sequences that formed stable G4-B (Figure 1E and Supplementary Table S1). Namely, these are G3B1, G3B2 and G2B2, where the first part of the term (‘GX’) refers to the number of intact G-stems, and the second (‘BX’) refers to the number of bulges. Our G4-B S models allow for a maximum of two bulges that may be located in one or two G-stems. In our tested pG4-BS sequences, we saw that the presence of contiguous cytosines and cytosine abundance is detrimental to G4-B formation (Figure 1D). Our results are supported by the observation that the presence of C bases in G4-S lower GC-skewness between the two DNA strands, ultimately promoting the formation of duplexes over G4s. The requirement of higher GC-skewness for stable G4 formation is one of the pillars of the scoring system used by Mergny *et al.* in their G4Hunter algorithm (39). In their work, cytosine clusters are more heavily penalized compared to stand-alone cytosines. To reduce the number of false-positive pG4-BS, we set up our search algorithm to filter out sequences with contiguous cytosines as we expect these sequences to prefer forming DNA duplexes over G4s. The final criteria for pG4-BS identification were to cap the maximum length of bulges and loops at three nucleotides. We restricted the length of loops to balance the potential loss of thermodynamic stability due to the inclusion of bulges with the higher stability conferred by shorter loops (44). In parallel, we used the traditional G4-S rules (four uninterrupted G-clusters of at least length 3, and a maximum loop length of 7) to identify pG4-CS in the human genome. The workflow used for pG4-BS and pG4-CS identification is shown in Figure 2A.

Using in-house Python search algorithms; we identified 1935686 pG4-BS and 954745 pG4-CS in the human genome. We removed sequences that overlapped between the two datasets and further excluded any sequence that co-localized with human repeat elements. After the filtering steps, we retained 245146 pG4-CS and 899687 pG4-BS sequences that constitute 25.7% and 46.5% of the originally identified sequences, respectively. Based on the differences in population size, our results suggest that pG4-BS represents an essential extension of potential sites of G4 formation in the human genome. Additionally, the majority (>65%) of the traditionally identified pG4-CS sequences overlap with one or more pG4-BS sequences. As such, the pG4-CS and pG4-BS could positively or negatively cooperate in their vicinity.

We further classified the 899 687 non-repeat overlapped pG4-BS according to the sequence model they match (Figure 2A). We observed that sequences corresponding to the G2B2 model were the most numerous, followed by those which correspond to the G3B1, and lastly the G3B2 model. We also compared the distribution of sequence lengths between pG4-BS and pG4-CS (Figure 2B). In the case of pG4-BS the most common sequence length was 20 nucleotides: 188626 (∼21.0%) of pG4-BS were of this length. For pG4-CS, the most common length was 24 nucleotides, with 23 766 (∼9.7%) sequences possessing this length. During our computational analysis, we allowed distinct G4-S to share up to three of their four G-tracts. However, we are unable to predict which of these overlapping G4-S would partake in biologically relevant G4-formation. To address this, we consolidated individual overlapping G4-S into the merged G4-S regions, which may contain multiple G4-S in proximity (Figure 2C and Supplementary Figure S1). This step resulted in 115 259 merged pG4-CS regions and merged 478263 pG4-BS regions. We also merged overlapping sequences in the model-specific pG4-BS data sets.

In the next step, we plotted the frequency distribution (FD) for each category of merged pG4-S regions to determine how the lengths are distributed for each dataset. As shown in Figure 2D, the FD functions for pG4-BS are more complex than those for the pG4-CS. The sequences belonging to the G3B1, G3B2 and G2B2 models show similar skewed FDs with similar modes, but different dynamic ranges for the region lengths for each of the three pG4-BS classes. Most of the merged pG4-S regions possess lengths below 30 nucleotides. Also, the negative slopes of the merged pG4-BS and merged pG4-CS are different. In the case of pG4-CS, the FD has a longer power law-like tail, suggesting a higher frequency of the longer regions.

We also observed that the length of the right tail of the FD belonging to each group varies; this feature correlates with the sample size of the sequence model. In particular, G2B2 sequences have the highest frequency of merged regions in the genome and have the longest FD tails. To account for this sample-size difference effect, we utilized same-size random samples (*n* = 63 660; the lowest number of regions among the studied categories) to test whether the FD of the lengths statistically differed between pG4-BS regions and pG4-CS regions. We observed significant differences between the lengths of regions belonging to any of the pG4-BS models and pG4-CS regions (K-S test, *P* < 2.20E–16). Additionally, the K-S statistic distance metrics between the paired distributions of same-size samples differ, showing the smallest differences between pG4-CS and non-model-specific pG4-BS regions (D = 0.447), followed by G2B2 type pG4-BS (*D* = 0.457), G3B2 type pG4-BS (*D* = 0.557) and G3B1 type pG4-BS (*D* = 0.664).

Our results suggest that the merged region length distribution is an essential characteristic that significantly differentiates our classes of G4-like DNA regions from pG4-CS. We do note that our search algorithm allows loops lengths of up to seven nucleotides for pG4-CS, while only up to three nucleotides for pG4-BS. These considerations were based on the expected negative association between loop length and thermodynamic stability of G4s (44).

### pG4-BS are present in the gene body or promoter region of >90% of protein-coding genes

Table 1 shows the distribution of pG4-BS and pG4-CS regions in the gene promoter (TSS) and the distinct gene segments in protein-coding genes (Ensembl 105). We note that G4-S that overlapped multiple gene segments were excluded, hence the counts in the table refer to unique mapping events for each given gene segment. Out of the 19 150 annotated human protein-coding genes, 94% (17 993 genes) contain at least one pG4-BS region in the gene body or promoter regions (2kb upstream of the TSS), while 76% (14 462 genes) overlap with at least one pG4-CS region in either sense or antisense orientation (Table 1 and Supplementary Tables S2.1 and S2.2). We also provide the number of protein-coding genes in which the associated pG4-BS regions co-localize with transcription factor binding sites (TFBS) and/or DNase-hypersensitive genomic regions. We refer to such sequences in the following sections as regulatory pG4-BS (Table 1).

**Table 1.** Counts of merged pG4-BS and pG4-CS uniquely mapping to different gene segments of protein coding genes in sense or anti-sense gene orientations

Region	Sense hits	Anti-sense hits	No. of gene symbols*	Proportion of genes	Ratio of gene proportions vs. pG4-CS
pG4-CS					
Genic or promoter	40 899	35 512	14 462	0.76	
Promoter	3504	3819	5089	0.27	
Gene body	37 725	32 022	13 603	0.71	
5′-UTR	941	1081	1758	0.09	
3′-UTR	1585	1780	2483	0.13	
Exons	671	1753	1720	0.09	
Introns	25 656	20 438	10 526	0.55	
pG4-BS					
Genic or promoter	154 252	131 742	17 993	0.94	1.24
Promoter	9860	10 855	10 483	0.55	2.06
Gene body	145 291	121 918	17 498	0.91	1.29
5′-UTR	2555	2345	3763	0.20	2.14
3′-UTR	4865	6609	6021	0.31	2.42
Exons	2961	4994	4404	0.23	2.56
Introns	102 960	81 617	15 094	0.79	1.43
Regulatory pG4-BS**					
Genic or promoter	102 831	88 778	17004	0.89	1.18
Promoter	8814	9723	9536	0.50	1.87
Gene body	94 835	79 991	16382	0.86	1.20
5′-UTR	2474	2279	3661	0.19	2.08
3′-UTR	3572	4867	4668	0.24	1.88
Exons	2156	3521	3368	0.18	1.96
Introns	63 835	50 731	13513	0.71	1.28

*Merged G4-BS which mapped to gene regulatory loci (DNAase hypersensitive regions and/or transcription factor binding sites).

**Human protein-coding genes (ENSEMBL 105).

Total number of annotated protein coding genes: *n* = 19 150.

Interestingly, 60% (11497/19150) of protein-coding genes overlapped with both pG4-BS and pG4-CS on the non-template strand, while 5295 genes (28%) were only pG4-BS positive, and only 466 (2%) genes were uniquely pG4-CS-positive (Supplementary Table S2.3). Similar numbers were observed when analyzing the presence of pG4-S on the template strand of protein-coding genes. In addition, we observed that ratios of the proportion of pG4-BS-positive genes compared to the proportion of pG4-CS-negative genes is >2 times larger in the 5′UTR, 3′UTR, exons and promoter regions (Table 1).

In summary, we identified 8807 pG4-BS-positive/pG4-CS-negative genes and mapped the positions of such pG4-BS genome-wide (Supplementary Table S3). Thus, the pG4-BS regions associated with these pG4-CS-negative (pG4-CS(–)) genes could represent a novel subset of potential gene regulatory signals that are separate from previously described pG4-CS. On the other hand, the high number of genes containing both pG4-CS (pG4-CS(+)) and pG4-BS (pG4-BS(+)) indicates that pG4-BS may extend the range of previously considered and biologically relevant G4 forming sites *in vivo*.

We note that our computational identification does not cover all potential forms of pG4-BS or pG4-CS. However, our selection criteria for both types of G4-S are based on experimental results that allowed us to develop a search strategy that can capture sequences that are most likely to be able to form stable bulged G4s *in vitro*.

The preferential occurrence of pG4-BS in promoters, 5′/3′UTRs, exons, and TTS boundaries suggests a regulatory role for pG4-BS in gene transcription initiation and splicing events

**Figure 3.**
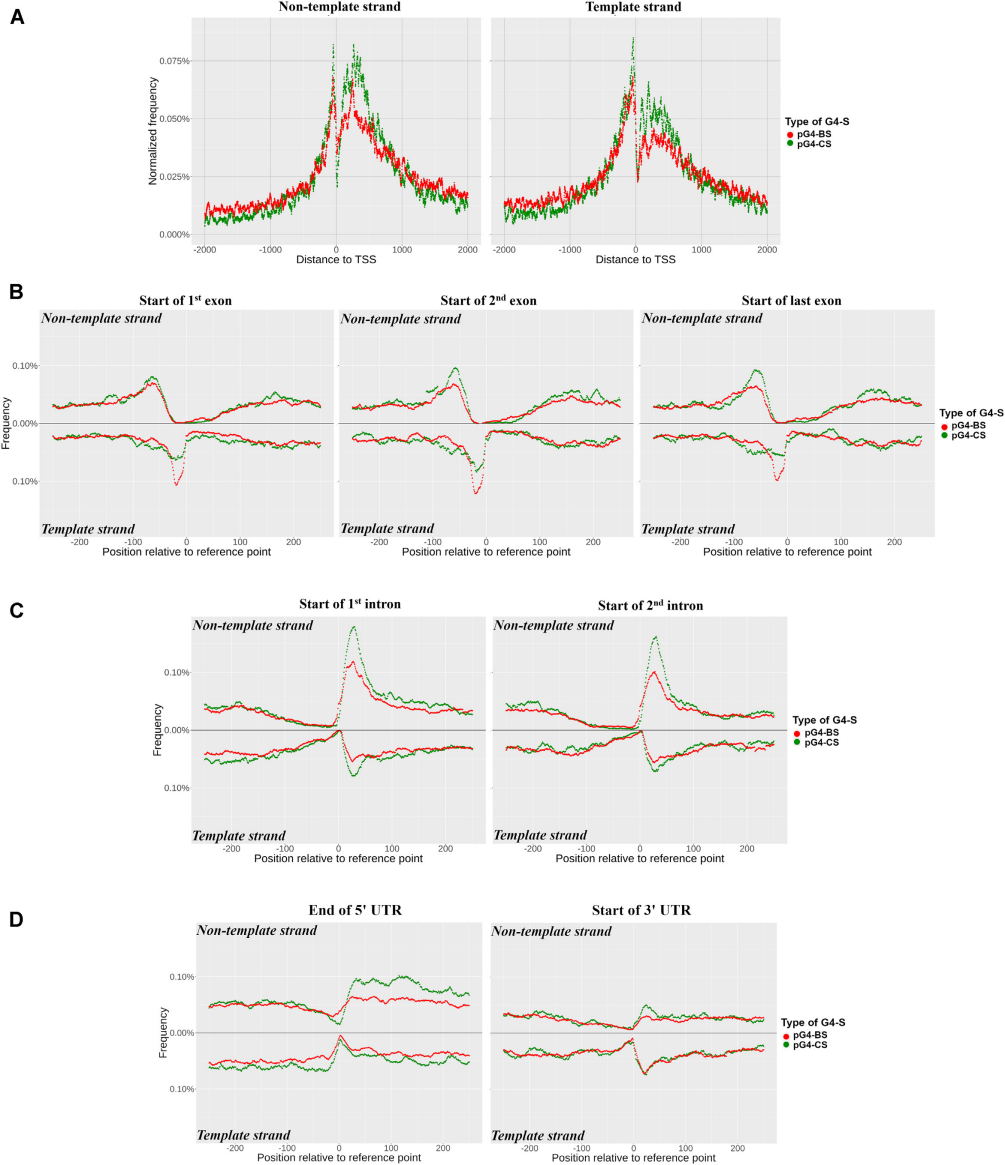
(**A**) Distribution of pG4-BS and pG4-CS in the proximity of the TSS of protein-coding genes. The frequency of individual pG4-S was counted at each given base position and normalized for the number of G4-S present across all positions are plotted over a ±2 kb region centered on the TSS. The frequency plots for the non-template and template strands are shown on the left and right panels, respectively. The TSS coordinates of the protein coding genes were acquired from Ensembl via the biomaRt tool. (B–D) FD functions of pG4-BS and pG4-CS around the start site of selected exons (**B**), introns (**C**) and 5′ and 3′ UTRs (**D**). Per nucleotide normalized frequency was calculated by counting the number of instances a given nucleotide was included in an overlapping pG4-S and was normalized to the total number of pG4-S occurrences across all positions. The plots show a ±200 bp region centered on the specific reference point.

In our previous analyses, we saw that pG4-BS are present in most human protein-coding genes. In our next step, we wanted to identify where pG4-BS regions frequently occur within a given gene. First, we counted the number of gene-associated G4-S that occurred at each nucleotide position around the TSS (±2 kb) of G4-S positive genes (Figure 3A). pG4-BS regions showed a bimodal asymmetrical FD of these sequences around the TSS (±2 kb), which is similar to that of pG4-CS regions. This suggests that pG4-BS could facilitate similar functions as pG4-CS, such as affecting the regulation of transcription and genome stability (13,67,68). We further analyzed the FD of pG4-BS occurrences in the proximity of the TSS, by decomposing the FD function into pG4-BS model-specific parts (Supplementary Figure S2).

The analysis of the distribution functions revealed that pG4-BS regions belonging to all three G4-BS models possess bimodal distributions. Out of our three G4-BS models, most of the pG4-BS regions that are localized near the TSS belong to the G2B2 model (Supplementary Figure S2a, b). However, the normalized plots reveal that G2B2-specific regions show similar density patterns as those belonging to the other models (Supplementary Figure S2c, d). These results suggest that pG4-BS regions could be enriched in either the proximal promoter (TSS) and/or at the end (TTS) of most of the protein-coding genes. The similarities between the distributions of pG4-BS and pG4-CS suggest the potential structural and functional roles of both.

We extended our analysis by determining the distributions of pG4-BS and pG4-CS regions at the start and end of distinct gene segments. For this analysis, we systematically studied the statistics of the following gene segments and their vicinity: TSS, termination site of 5′ UTR, initiation sites of 1st, 2nd and last exons, initiation sites of 1st, 2nd and last introns, initiation site of 3′ UTR, and the transcription termination site (TTS) of the gene. As above, we calculated the per nucleotide frequency of pG4-BS and pG4-CS regions around (±2*k* bases) the selected gene segments. We note that G4-S overlapping multiple gene segments were included in all associated categories. Our results show that pG4-BS and pG4-CS regions show similar distribution patterns across the studied boundaries of gene segments. Interestingly, pG4-BS regions are more abundant compared to pG4-CS regions, which can be due to the much higher pG4-BS presence in protein-coding genes that we observed earlier (Table 1). Despite the general similarities, we did note differences between the enrichment of pG4-BS and pG4-CS regions following normalization, especially in the studied exons and introns regions near gene segment boundaries (Figure 3b-c). On the non-template strand, pG4-CS regions were more enriched in the vicinity of the initiation sites of the 1st and 2nd exons, and the initiation site of the 1st intron. On the template strand, the initiation sites of the 1^st^ and 2nd exons showed stronger enrichment for pG4-BS regions. This indicates that pG4-BS may have strand-specific functions that differ from those held by pG4-CS. We also observed periodicity in the distribution functions of the G4-S regions around the intron–exon junction sites. The studied exons showed enrichment of G4-S (both pG4-BS and pG4-CS) upstream of the transcription initiation site (TIS), followed by a marked absence of G4-S in the immediate vicinity of the initiation site on both non-template and template strands (Figure 3B). On the other hand, studied introns showed weak G4-S presence upstream of their initiation site, followed by an enrichment of pG4-S in the area downstream (Figure 3C). We also observed an increase in pG4-BS and pG4-CS presence downstream of the end site of the 5′UTR and at the start of the 3′ UTR (Figure 3D).

We further analyzed the G4-BS-model FD of pG4-BS regions across the same gene segments. pG4-BS regions comprised of G2B2 type pG4-BS were most common, as we observed before around the TSS (Supplementary Figure S2). Following normalization, we saw that the three models are similarly enriched in the studied genomic regions, with the main differences observed at sites of major pG4-BS region presence (e.g. initiation sites of exons and intron). On the non-template strand, G3B1 type pG4-BS regions showed the strongest enrichment at the initiation sites of the 1st and 2nd intron, followed by G3B2 and G2B2 type regions. On the template strand, G3B2 type regions were slightly more enriched than G3B1 type ones at the initiation sites of the 1st and 2nd exons. These results suggest that G4-B arising from pG4-BS with different sequence compositions could possess strand- and/or gene segment-specific functions.

### Modeling the distribution of pG4-BS count per gene

**Figure 4.**
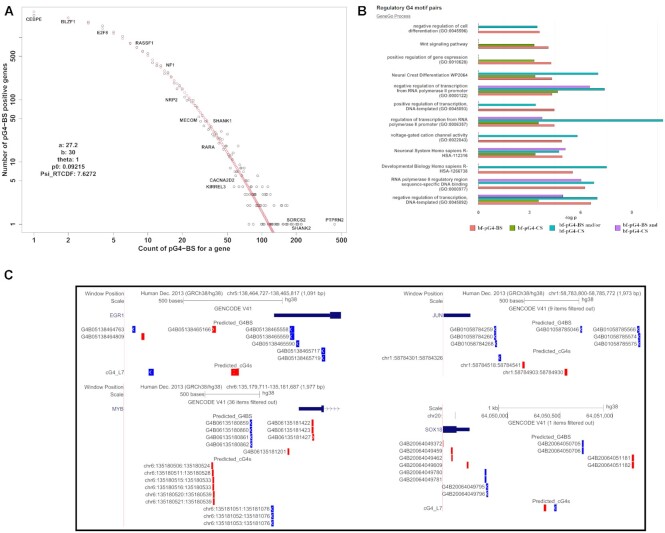
(**A**) Log–log plot of the distribution of pG4-BS count per protein coding genes. Selected gene symbols are indicated at the positions corresponding to their relative count of pG4-BS. Our best fit model was based on the Kolmogorov-Waring function with parameters θ = 0.99, α = 27.2, β = 30. (**B**) Gene ontology analysis of promoter-associated regulatory bf-pG4-BS and bf-pG4-CS in human protein coding genes. We chose examples of GO terms that are both significant (adj. *P*-value < 0.05) and were observed in at least 2 of the tested gene sets. The *P*-values are presented as negative logarithms. (**C**) Examples of bf-pG4-BS located in the promoter regions of the EGR1 (top left), JUN (top right), MYB (bottom left) and SOX18 (bottom right) genes.

We present a log-log plot of the empirical frequency distribution (EFD) of pG4-BS region counts per protein-coding genes in Figure 4a and highlight the frequency of occurrence of pG4-BS for several essential cancer-associated genes and the genes considered in this study. Here we focused on the pG4-BS that are located in the promoter (2 kb upstream of the TSS) and the gene body on the non-template strand. The EFD of pG4-BS counts per protein-coding genes has a long right tail indicating that few genes contain a high abundance of pG4-BS, while most others are sparsely populated. Such EFDs are often driven by the Kolmogorov stochastic birth-death processes (BDPs) and are commonly found in complex, interconnecting evolving biosystems (69,70). We found that the distribution of pG4-BS in protein-coding genes is a skewed probability distribution function (PDFs) with a power-law-like right-side tail (Figure 4A). Our best-fit model was the Kolmogorov-Waring PDF (69,70) with estimated parameters θ = 0.99, α = 27.2, β = 30 (see Supplementary Methods) (69,70). This model proposes stationary BDP evolving at a balance of the gain and loss events (i.e. pG4-BS in a gene). Using this approach allows us to get information about the probabilistic mechanisms leading to the skewed form of the EFD and compare the system-wide effects of different conditions on the G4-BS landscape. In particular, these estimates allowed us to calculate the probability of a ‘non-observed event’, called ${p_0}\ (\ {p_0} = \ 1 - \hat{\alpha }{\rm{\ }}/{\rm{\hat{\beta }}}$). Based on the curve fitting (${p_0}$= 0.0925), we expect that our algorithm captures over 92% of the pG4-BS that could be observed in the human genome.

### Gene ontology analysis of genes encompassing pG4-BS

To investigate the potential regulatory roles of pG4-BS across specific biological classes of genes, we carried out GO analysis for the group of protein-coding genes that contain at least one pG4-BS region (*n* = 17 993) in their promoter (region 2 kb upstream of TSS) or their gene body. First, we utilized the database for annotation, visualization, and integrated discovery (DAVID) bioinformatics resources using all annotated genes as the background. We observed strong tissue specificity (*P*-value <1E–5, after Benjamini correction), with the strongest association observed with brain tissue (Supplementary Table S4). Specifically, out of the 17 993 pG4-BS(+) genes, 7319 (40.6%) are associated with the brain tissue (termed ‘brain’ under the UP_TISSUE annotation set of DAVID bioinformatics resources). In addition, we found 46 terms enriched (*P* < 1E–5, after Benjamini correction), many of which were associated with transcriptional regulation (e.g. ‘positive regulation of transcription from RNA polymerase II promoter (GO:0045944)’), or with cell signaling pathways (e. g. ‘MAPK signaling pathway’, and ‘Pathways in cancer’).

Next, we investigated if the degree of pG4-BS presence shown by the skewed EFD (Figure 4a) corresponds with specific biological classes of genes. To test this, we randomly chose the same-sample size gene subsets (*n* = 1000) from the beginning of the EDF (‘low’ pG4-BS content), the tail of the EDF (‘high’ pG4-BS content) and the area between these two (‘intermediate’ pG4-BS content). Among the three gene sets, we observed strong tissue specificity (adj. *P*-value < 1E–5, after Benjamini correction) with brain tissue in the case of the ‘high’ gene set. We also ran our gene lists through the Enrichr gene set enrichment tool to detect statistically significant enrichment of biological terms. We observed strong statistical significance with GO terms only in the case of the samples with ‘high’ pG4-BS content (data not shown).

We also analyzed the potential roles of pG4-BS in the regulation of transcription across different functional classes of genes by performing GO analysis on the subset of pG4-S(+) genes that contain at least one regulatory pG4-BS and/or regulatory pG4-CS region in their promoter region (2kb upstream of the TSS) (Supplementary Table S5.1). In the case of pG4-BS(+) genes, we observed a statistically significant enrichment (adj. *P*-value < 0.05) in 252 terms from the included databases (Supplementary Table S5.2). In the case of regulatory pG4-CS, we identified 102 enriched terms (adj. *P*-value < 0.05) (Supplementary Table S5.3). Between the two gene sets, 75 of the enriched terms were common. Interestingly, when we repeated the analysis using genes with only on type of pG4-S or both, we observed the strongest enrichment in terms of genes associated with both types of regulatory pG4-S (Supplementary Tables S5.4–S6). The top enriched terms were associated with signaling pathways (e.g. Ras signaling pathway, PI3K-Akt signaling pathway) (in addition, see Supplementary Files 1.2.1).

GO analyses were also carried out separately for genes with promoter-associated regulatory pG4-BS located on either the non-template strand (n = 3165), template strand (*n* = 3610), or both (*n* = 2761) (Supplementary Table S6.1). For our functional analyses of these pG4-BS-positive gene subsets, we used triplicates of 1000 randomly sampled genes from the gene subsets. We observed strong enrichment (adj. *P*-value < 0.05 in at least one of the triplicates) of 73 terms in the case of genes with regulatory pG4-BS located on both non-template and template strands. Among the results were terms related to the development and cellular differentiation, as well as those related to the regulation of transcription (e.g. ‘positive regulation of transcription, DNA-templated (GO:0045893)’ (Supplementary Table S6.2). We observed weak or no significant enrichment in genes with single-strand pG4-BS presence.

Additionally, we conducted pathway enrichment analysis using the list of genes containing regulatory bf-pG4-BS in their promoters (*n* = 2761) as input for the KEGG Pathway Database (2021). Amongst the enriched pathways (Supplementary Figure S3a-b; Supplementary Table S6.3), we observed signaling pathways that have been linked to diseases, such as different cancers, and neurodegenerative and cardiovascular diseases (71–73). We saw similar enriched terms when using another pathway database (WikiPathways 2021). Using the String database web tool (version 11.5), we constructed the protein-protein interaction network of genes associated with at least one of the pathways shown in Supplementary Figure S3a or S3b (Supplementary Figure S3c). We observed a significant degree of overlap between the genes associated with the selected pathways (Supplementary Figure S4).

### New gene regulatory signals are defined by back-forward paired pG4-BS (bf-pG4-BS)

Prompted by our observation that simultaneous pG4-BS presence on both *non-template and template strands* resulted in the high-significance enrichment of more biological terms, we performed an in-depth characterization of these genes (Supplementary Table S6.1).

First, we did not observe significant differences in the distribution of relative exon count frequencies between our gene sets (Supplementary Figure S5a). In all three cases, the exon counts showed a skewed FD, with a long right tail. This indicates that most of the genes in the three gene sets possess lower exon counts (<25), while few contain much higher numbers of exons. Additionally, we looked at how the genes in the different gene sets are distributed amongst the 24 human chromosomes (Supplementary Figure S5b). We did not observe a strong bias for any chromosome in any of the three gene sets. On most chromosomes, the percentage of genes with pG4-BS regions on both strands was less compared to genes with either non-template or template pG4-BS regions. In addition, we did not observe a significant correlation between the number of genes and their flank regions containing bf-pG4-BS genome architecture and the size of the chromosome they are located on (Kendall's tau: 0.21, *P*-value = 0.178) (data not shown). This indicates that the presence of bf-pG4-BS is likely not due to random chance, but rather appears in the genome due to evolutionary pressure and likely fulfills important biological roles.

To determine if our findings are pG4-BS-specific, we carried out a similar analysis only for genes with promoter-associated regulatory pG4-CS. As before, these regulatory pG4-CS were either located on only a single strand only (non-template or template, *n* = 1870 and 2120, respectively) or both strands in a back-forward (bf) genome architecture (*n* = 698). Here, we took triplicate random samples of 300 genes for every gene set and performed GO analysis. Using same-size random samples, we observed similar trends, with bf-pG4-CS containing genes being the only gene set enriched for biological terms (data not shown).

Our next question was whether the strand-specificity of pG4-S localization resulted in different biological functions and molecular mechanisms. To answer this question, we carried out a comparative gene set enrichment analysis of genes with regulatory G4-S on one or both DNA strands in the promoter region (Supplementary Table S6.4). Our results showed the statistically significant enrichment of 71 biological terms for these genes (Supplementary Table S6.5), with most related to the nervous system, development, transcriptional regulation, signaling pathways, and pathways in cancer.

We confirmed our findings by utilizing exclusive and same-sized random samples of genes with either bf-pG4-BS, bf-pG4-CS or both in their promoter regions (Supplementary Table S6.6). Interestingly, while some terms we enriched in gene sets with only one type of bf-G4-S, terms previously identified (e.g. nervous system, transcriptional regulation) were only enriched in genes with both types of bf-G4-S in their promoters (Figure 4B and Supplementary Table S6.7). This suggests that different types of bf-pG4-S may be acting in concert and that simultaneous presence on both strands may help facilitate the biological functions of G4s. Examples of genes with both bf-pG4-BS- and bf-pG4-CS-positive promoters are presented in Figure 4C.

### High-throughput mapping of potential G4s in the human genome confirms the existence of a large family of stable G4-B structures

Here, our goal was to assess how well the results of our computational prediction aligned with those produced by current experimental methods developed for the genome-wide detection of G4-S. G4-seq is a high-resolution next-generation sequencing (NGS) method that was developed to detect putative G4 forming DNA sites in the human genome by Chambers et al (74) and later improved by Marsico *et al.* (65). The latter study provided data sets of experimentally detected G4 sites for several species, including humans. We downloaded the associated data sets from the Gene Expression Omnibus (GEO) (accession number GSE110582) and preprocessed them as described in Methods.

First, we analyzed the FD of the lengths of G4-seq regions. We plotted the FD of G4-seq region lengths alongside those of pG4-BS and pG4-CS regions (Supplementary Figure S6a). We also included a subset of G4-seq regions, termed ‘higher confidence’ G4-seq regions. This group is comprised of genomic sites where G4 presence was detected under both types of stabilizing conditions (K^+^ or K^+^ and PDS) in the G4-seq experiments. We observed that the G4-seq region length distribution functions show a unimodal distribution in all cases, and they are fitted well by the log-normal function or Weibull function (Supplementary Figure S6a). However, the respective peaks of each distribution (and the corresponding most common sequence length) are markedly different between computational (pG4-BS: 20 nucleotides; pG4-CS: 25 nucleotides) and experimental data (‘pooled’ G4-seq: 135 nucleotides; higher confidence G4-seq: 120 nucleotides). This was also true when looking at other basic statistics of these datasets (Supplementary Table S7.1a)

**Figure 5.**
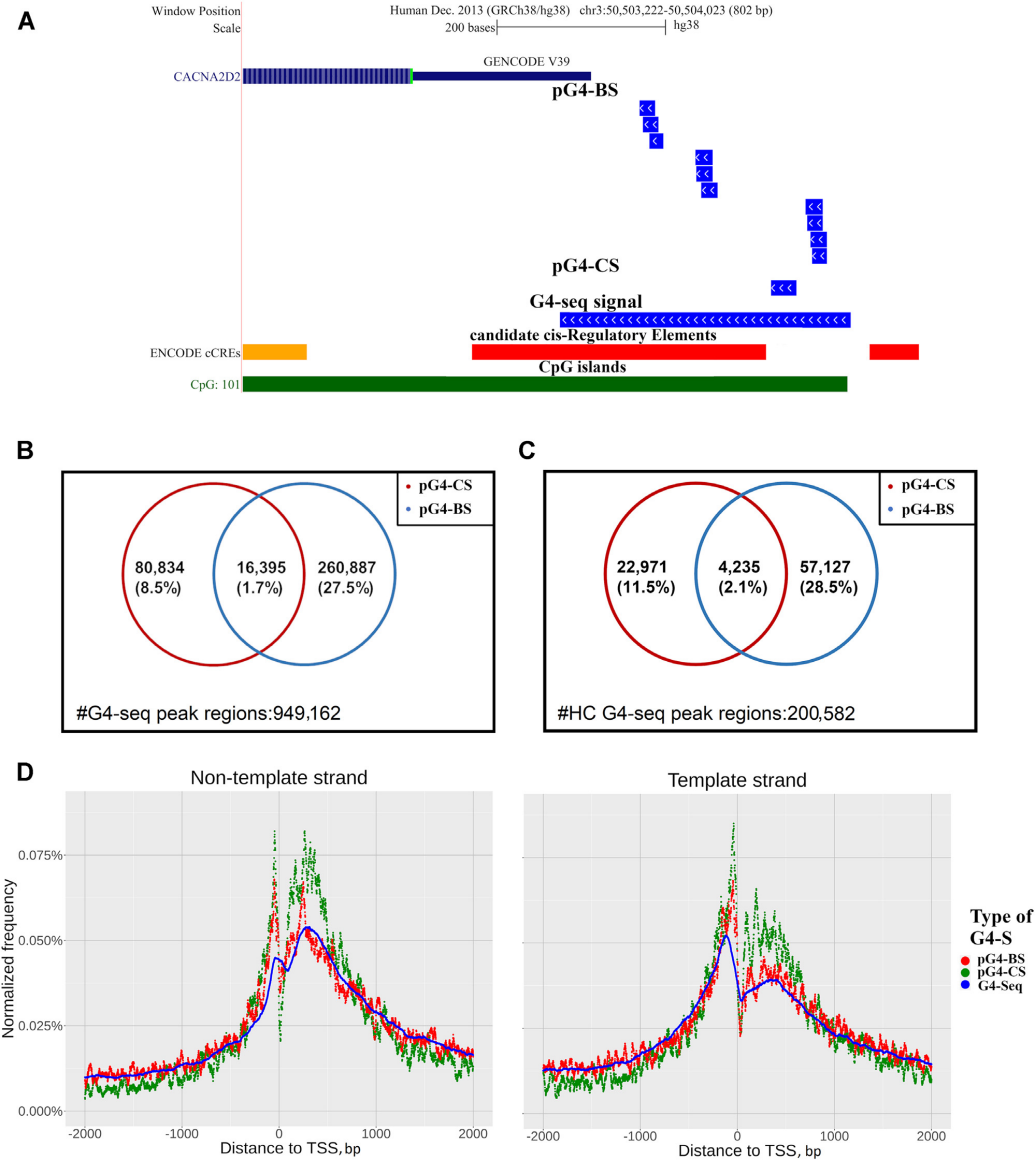
(**A**) UCSC genome browser image of the promoter region of the CACNA2D2 gene. Tracks show that predicted pG4-BS regions overlap with experimental G4-seq signals, ENCODE cCREs and CpG islands. The image shows that computationally predicted pG4-BS regions are embedded in the G4-seq region. Additionally, our predicted regions more accurately pinpointing potential sites of G4 formation, compared to G4-seq. (B, C) Decomposition of experimentally detected G4-seq regions. (**B**) Number of non-repeat overlapped experimental G4 regions (*n* = 895 179) supported by pG4-BS and/or pG4-CS regions. (**C**) Number of non-repeat element overlapping experimental G4 regions (*n* = 192 448) that are both protein-coding gene associated and overlap gene regulatory loci (transcription factor binding sites and/or DNase I hypersensitive sites) and are supported by pG4-BS and/or pG4-CS regions. (**D**) FD functions of G4-seq, pG4-CS and pG4-BS regions around the TSS of protein-coding genes. The FD of individual G4-S counts at each given base position, normalized for the number of G4-S present across all positions are plotted over a ±2 kb region centered on the TSS for the non-template strand (left) and the template strand (right).

We further analyzed the distribution of the length in the context of overlaps with pG4-BS belonging to each of our G4-BS models (Supplementary Figure S6b). The normalized FD showed similarity the FDs of the models. To further evaluate the sensitivity of the G4-seq method, we compared the FD of the percentage of guanine and cytosine bases (GC%) of G4-seq, pG4-BS and pG4-CS regions with those of G4-seq regions. In every case, we randomly selected 10 000 random sequences for comparison and calculated the frequency of GC% values for each sample (Supplementary Figure S6c). The difference of mean GC% was statistically significant in each of the G4-BS models and pG4-CS when compared to those of G4-seq regions (Wilcoxon Paired test, *P* < 2.2e–16). Additionally, the differences between the FDs of G4-S samples and G4-seq regions were also statistically significant in all cases (K–S test, *P* < 2.2e–16). Overall, our findings indicate that the length of the G4-seq regions is generally longer and has lower GC% compared to pG4-BS and pG4-CS. Due to the length of G4-seq regions, the determination of the exact site of G4 formation becomes difficult (Figure 5A). Additionally, the presence of multiple overlapping G4-S further complicates the deeper classification of G4-seq signals.

Despite these complications, our comparison of pG4-BS and pG4-CS mapping to G4-seq regions revealed that 35.9% of G4-seq regions are supported by either pG4-BS, pG4-CS or both (Figure 5b and Supplementary Table S7.2). The number of G4-seq regions supported by only pG4-BS was more than three times the number of pG4-CS(+) G4-seq regions (260997 vs. 80834). Out of the 260997 pG4-BS(+)/pG4-CS(–) G4-seq regions over half (57%) were supported by pG4-BS belonging to the G2B2 model (data not shown). Next, we performed the same analysis using subset of ‘higher confidence’ G4-seq regions. Out of these 200 582 G4-seq regions, 42% were supported by either pG4-BS, pG4-CS or both, indicating a minor enrichment in the number of supported G4-seq regions in this subset (Figure 5c and Supplementary Table S7.2). Our findings highlight that pG4-BS provide a potentially major extension and may be able to explain a significant portion of pG4-S detected by G4-seq. We note that over 50% of detected G4-seq regions are not covered by either type of G4-S, suggesting the existence of other G4 structures, some of which have already been reported (i.e. duplex stem-loop containing G4s) (45) and the existence of G4-seq regions that are false positive.

As an additional step to ensure that our computational predictions are well-founded and persuasive, we analyzed how well G4-seq-derived data supports our pG4-BS dataset. We utilized same-sized samples of pG4-BS and background (random) sequences and calculated the proportion of ‘pooled’ G4-seq data-supported sequences in each sample (Supplementary Table S7.2). Based on the proportions of ‘pooled’ G4-seq supported sequences in pG4-BS data and the associated background sample, we calculated the enrichment score (ES = 17.37, *P*-value < 0.001). We repeated the analysis using a random sample of pG4-CS and corresponding background (random) sequences and observed a similar trend (ES = 18.67, *P*-value < 0.001). Taken together, these results indicate that G4-seq signals are similarly enriched in pG4-BS as in pG4-CS, lending further support for the validity of our method and findings.

Next, we analyzed the FD of ‘pooled’ G4-seq, pG4-CS and pG4-BS regions around the TSS of protein-coding genes (Figure 5D). We observed that the distribution of G4-seq regions did not follow the pG4-BS or pG4-CS distributions at the single-nucleotide level. We hypothesize that the longer regions detected by G4-seq are inadequate to fully differentiate the peaks of the distribution function, which is where G4-S are most commonly found in the studied genes. To underline these findings, we calculated and plotted the EFD of G4-seq regions for specific gene segments, alongside pG4-CS and pG4-BS regions (Supplementary Figure S7). In every case, our results showed that computational prediction of G4-S provides better resolution for genome-wide mapping of putative sites of G-quadruplex formation. Furthermore, pG4-BS underlie a significant portion of pG4-CS(-) G4-seq regions, corroborating the results of our computational genome-wide prediction.

### Majority of reproducible G4 CUT&tag peaks are supported by pG4-BS

To assess the validity of our computational predictions, we also utilized data derived from G4 CUT&Tag experiments (5). We downloaded and processed the raw data as described in Materials and Methods. Initial analysis of G4 CUT&Tag signal coverage and peak region positions showed both common (Supplementary Figure S8a) and unique peaks (Supplementary Figure S8b) between the tested cell lines. We observed, however, that the sequence library sizes varied among the samples, as shown in Supplementary Table S7.1b. In addition, there is no saturation of the read count in any of the libraries. A larger library sample size results in more significant peak regions in the libraries. In addition, we saw significant variation between the numbers of peaks called for each cell line. Importantly, between samples of the same cell line (HEK293T) we observed only 40% of commonly identified peaks. The low reproducibility of peak regions between HE293T cell line samples, the lack of replicate samples in other cell lines, and significant sample size variation made it necessary for us to treat available data as six DNA samples from the human genome. Using this approach, we derived a combined dataset, where we pooled any G4 regions (peaks) detected in at least one of the samples (*n* = 45 968) (Materials and Methods).

To start, we compared the lengths of CUT&Tag peaks to those of computationally predicted and G4-seq derived regions (Supplementary Figure S6a). We observed that the ‘pooled’ set of CUT&Tag peaks possessed lengths longer than pG4-BS, pG4-CS and G4-seq regions (Supplementary Table S7.1a). Such differences in sequence length indicate varying resolutions between these methods, complicating the direct comparison of datasets.

**Figure 6.**
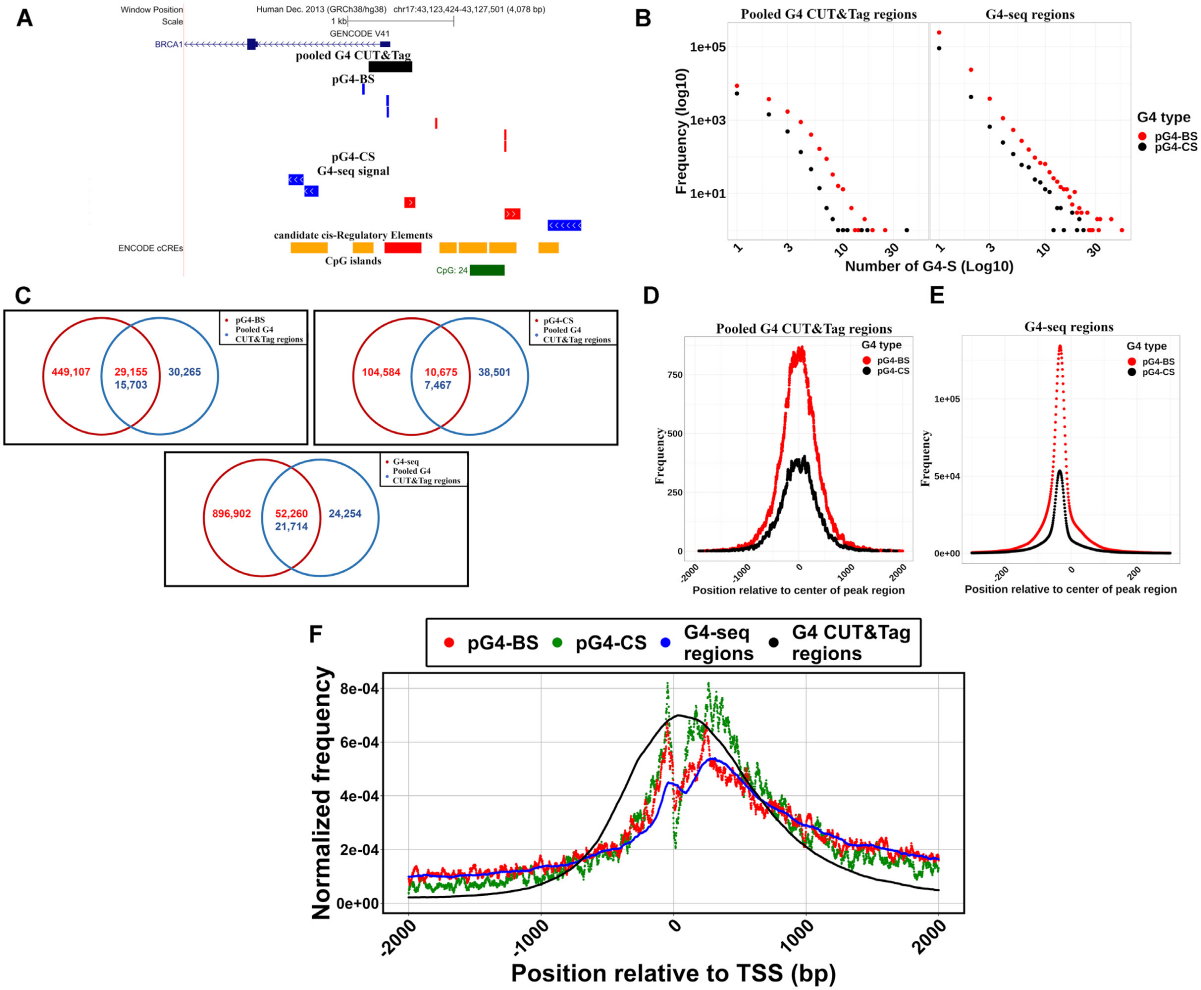
Analysis of the association between computationally predicted and experimentally detected native G4 forming sequences. (**A**) Genome browser image of a G4 CUT&Tag region located in the BRCA1 gene promoter. This region contains pG4-BS, but no pG4-CS. (**B**) FDs of the number of pG4-BS and pG4-CS overlapping G4 CUT&Tag or G4-seq regions. The axes have been log10 transformed for visualization purposes. (**C**) Venn diagrams showing the number of overlapping regions between the pooled G4 CUT&Tag dataset and pG4-BS, pG4-CS and G4-seq. Since one-to-many associations are expected, the numbers in the intersection of the sets are color-coded and indicate the number of overlapping regions in each dataset. (D, E) Plots indicating the most common location for pG4-BS and pG4-CS about the center point of G4 CUT&Tag (**D**) and G4-seq regions (**E**), respectively. (**F**) FDs of common sites of G4 occupancy around the transcription start site (TSS) according to pG4-BS, pG4-CS, G4-seq regions and G4 CUT&Tag regions.

Next, we looked at the association between our computational predictions and G4 CUT&Tag regions and observed instances of many-to-one mapping (Figure 6A). To test, whether the longer sequence lengths of G4 CUT&Tag and G4-seq regions can be explained due to the presence of pG4-S clusters, we determined the FD of the number of computational motifs associated with each type of sequences (Figure 6B). Based on our observations, most G4 CUT&Tag and G4-seq regions overlap with one or few (<3) pG4-S, so the observed differences in sequence lengths cannot be attributed to underlying pG4-S clusters. As the next step of our analysis, we determined the number peaks supported by either computational (pG4-BS/pG4-CS) or experimental (G4-seq) predictions in the CUT&Tag dataset (Figure 6C and Supplementary Table S7.3). We observed that in the pooled CUT&Tag dataset (*n* = 45 968), 17802 (38.7%) peaks were overlapped with pG4-BS, pG4-CS or both. To determine where the overlapping pG4-S motifs are commonly located in experimentally derived G4 regions, we mapped pG4-S to both G4 CUT&Tag and G4-seq regions and identified the most common positions in relation to the center of the given experimental G4 region (Figure 6D and E). In the case of the CUT&Tag data, we saw that both pG4-BS and pG4-CS are roughly uniformly distributed around a 2000 bp region around the center of CUT&Tag regions. For G4-seq regions, computational motifs are more concentrated and are most commonly located upstream of the center of the region. In both datasets regions are more commonly associated with pG4-BS over pG4-CS.

Next, we utilized the same random-sampling approach as for G4-seq: briefly, we selected 10 000 random computationally predicted sites of G4-formation (pG4-BS or pG4-CS) and created corresponding background samples, while controlling for sequence length and chromosomal location. Then, we calculated the number of overlaps between each of our G4-S and background samples and the pooled G4 CUT&Tag peaks (*n* = 45 968). Overall, we observed similar enrichment of both pG4-BS (ES = 6.1) and pG4-CS (ES = 7.5) over their respective backgrounds in the pooled dataset (*P*-value < 0.001) (Supplementary Table S7.3). This suggests that both types of computationally predicted G4-S are more strongly associated with experimentally derived G4-sites than expected due to random chance. While these results are expected for pG4-CS, the fact that we observed similar outcomes for pG4-BS underlines their relevance as biologically significant G4-like structures.

Finally, we overlayed the distribution of G4 CUT&Tag regions around the TSS of protein coding genes with those of pG4-BS, pG4-CS and ‘pooled’ G4-seq regions (Figure 6F). We observed that the resolution of the CUT&Tag data is indeed lower, apparent from the lack of two distinguishable peaks observed for both computationally predicted motifs and G4-seq regions. However, despite the limitations in resolution, the overlapping between the distributions indicate that G4 CUT&Tag regions are preferentially detected in the regions around the TSS where computational predictions and G4-seq regions have been identified. Overall, these findings establish mutual support between our predictions and experimentally derived G4 sites.

### Start and end sites of RLFS show strand-specific enrichment of pG4-S, suggesting the roles of pG4-S in the initiation and termination of RNA:DNA hybrid formation

**Figure 7.**
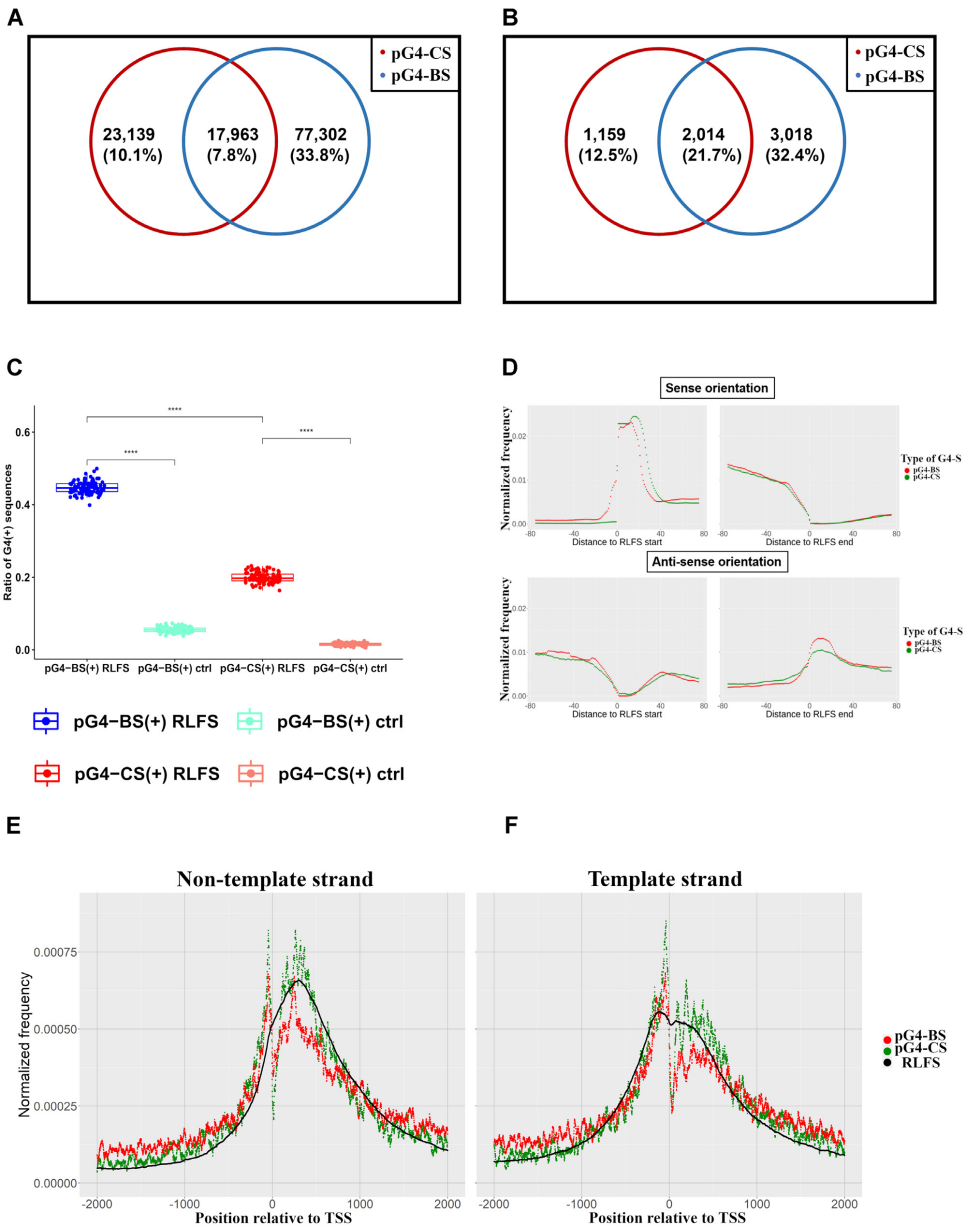
(**A**) Absolute numbers and percentages of human RLFS clusters (*n* = 228 999) supported by either pG4-CS regions, pG4-BS regions or both (left panel). (**B**) Absolute numbers and percentages of promoter-associated RLFS clusters (*n* = 9304) supported by either pG4-CS regions, pG4-BS regions or both (right panel). A minimum overlap of one nucleotide was required between coordinates to establish support in the case of both G4-S. (**C**) Ratio of pG4-BS(+)/pG4-CS(+) RLFS and background sequences. Each data point indicates the ratio of G4(+) sequences in a given sample. Overall, we selected 100 samples, each containing 1000 randomly selected RLFS/background regions. We statistically compared the samples using the Mann–Whitney *U* test. Color codes as follows: blue = ratios of pG4-BS(+) RLFS in RLFS samples, light blue = ratios of pG4-BS(+) background sequences in background samples, red = ratios of pG4-CS(+) RLFS in RLFS samples, light red = pG4-CS(+) background sequences in background samples. (**D**) Relative FD of pG4-BS and pG4-CS regions around the start and end sites of RLFS. Using K–S tests, the distributions are statistically different (*P* < 2.2e–16) between pG4-BS and pG4-CS in all cases. However, the Distance metric is different for different strands and sites. Start of RLFS on non-template strand, *D* = 0.1205. End of RLFS on non-template strand, *D* = 0.0368. Start of RLFS on template strand, *D* = 0.0372. End of RLFS on template strand, *D* = 0.0735. (E, F) FD functions of R-loop forming sequence (RLFS) clusters, pG4-CS and pG4-BS regions around the TSS of protein-coding genes on the non-template (**E**) and template strands (**F**). The FD of individual G4-S counts at each given base position, normalized for the number of G4-S present across all positions are plotted over a ±2 kb region centered on the TSS.).

The co-occurrence of G4(s) and R-loops may be associated with increased DNA damage, genome instability and cancer (14,51,75). For instance, it was found that G4 stabilizing ligands can cause R-loop mediated DNA damage (60). However, to date, no studies have analyzed the relationship between G4-like sequences and R-loops in a systematic manner. We found that among merged RLFS clusters in humans (*n* = 228 999) over 50% overlap with pG4-BS, pG4-CS or both (Figure 7A). However, uniquely pG4-BS(+) RLFS were three times as common as RLFS clusters with only pG4-CS support. In the subset of RLFS clusters associated with promoters (located within 2kb upstream of the TSS; *n* = 9304), we saw slightly higher ratios of uniquely pG4-CS(+) RLFS (10% versus 13%), and a lower percentage of pG4-BS(+) RLFS (34% versus 32%) (Figure 7B). Interestingly, we observed a major increase in the percentage of RLFS with both pG4-BS and pG4-CS support in this subset (8% versus 22%).

Next, we used a bootstrapping approach to select 100 samples of 1000 randomly selected RLFS regions and the same number of random background sequences. During the sampling of background sequences, we controlled for the length and chromosomal location, thereby eliminating chromosome size and sequence length-associated biases. In each of the RLFS and background samples, we determined the ratio of sequences overlapping with pG4-BS and pG4-CS in separate analyses. Our results revealed a statistically significant difference (Mann–Whitney U test, *P*-value < 0.001) between the ratios of pG4-BS(+)/pG4-CS(+) RLFS versus background sequences (Figure 7C). Furthermore, the ratio of pG4-BS(+) RLFS was also significantly (Mann–Whitney *U* test, *P*-value < 0.001) higher compared to the ratio of pG4-CS(+) RLFS. Overall, this suggests that pG4-BS and pG4-CS are more commonly embedded in RLFS regions than we’d expect due to chance. Promoter-associated RLFS are well-known transcriptional regulators and are reported to frequently co-localize with pG4-CS (54). Our findings extend these observations to pG4-BS and show an even stronger association between RLFS and pG4-BS, compared to pG4-CS. Statistical testing supports (Fisher's test, *P*-value < 0.001) our hypothesis that the co-localization of same strand RLFS and pG4-S (both pG4-BS and pG4-CS) in promoters are non-random, and that these DNA structures may constitute parts of a higher-order structural and functional unit.

Next, we analyzed the EFD of pG4-BS and pG4-CS regions over the start and end sites of RLFS clusters (Figure 7D). Overall, 34% (77345) and 13% (30793) of the 228 999 RLFS clusters overlapped with at least one same-strand G4 region (pG4-BS or pG4-CS) near the start site and end site, respectively. The FDs show that both pG4-BS and pG4-CS regions are enriched immediately downstream of the start site of the RLFS (the R-loop initiation zone, RIZ) and are mostly absent after the end site in the sense orientation. In the gene antisense orientation, pG4-BS and pG4-CS regions are more common downstream of the end site of RLFS. To further characterize the relationship between RLFS and G4-S, we calculated the per nucleotide FD of pG4-BS, pG4-CS and RLFS around the TSS-proximal regions of protein-coding genes. We observed strong co-localization between RLFS and both types of G4-S on both non-template (Figure 7E) and template strands (Figure 7F). Overall, these results indicate that G4 structures (pG4-BS and pG4-CS) could possess strand-specific roles in the initiation, stabilization and termination of RNA:DNA hybrid formation, and may further function as boundaries of R-loop formation regions.

### Structural studies confirm the ability of pG4-BS to form G4-B structures is dependent on sequence characteristics

To validate the ability of our pG4-BS models to identify sequences that can form G4s in human genes and gene regulatory regions, we consider 38 pG4-BS sequences that were mostly identified in proximal promoter regions of randomly selected cancer-associated genes (Supplementary Table S8). Among these 38 sequences, 32 sequences were represented by pG4-BS following the characteristics of our pG4-BS models, while remaining six sequences have one or more properties that prevent a formation of stable G4-conformation based on our models (Methods, Supplementary Table S8.1).

**Figure 8.**
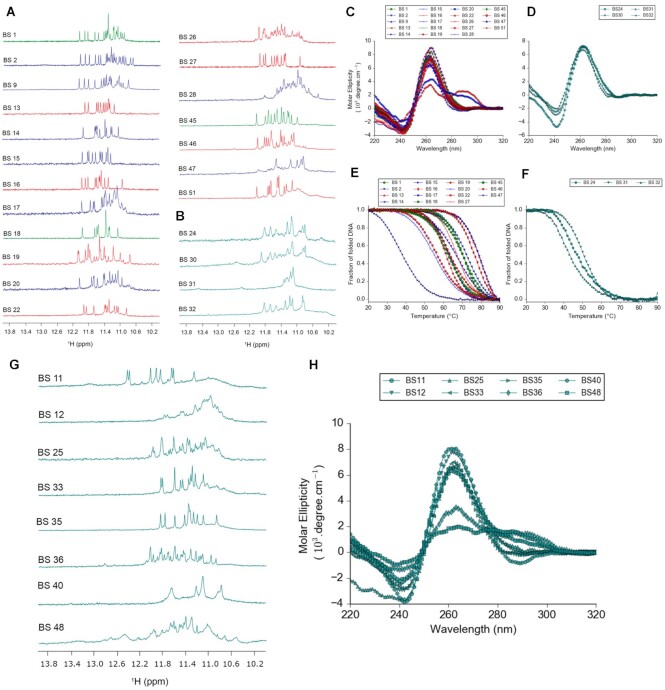
(A–F) Thermodynamic analysis of pG4-BS with a single bulge with varying bulge lengths. (**A**) The majority of NMR spectra belonging to sequences with single nucleotide bulges indicate the formation of a single dominant G-quadruplex conformation. (**B**) NMR spectra of pG4-BS with multiple nucleotide bulges suggest the lack of a stable G4 conformation. (**C**) CD spectroscopy result of pG4-BS with one single nucleotide bulge. (**D**) CD spectroscopy result of pG4-BS with one multiple nucleotide bulge. (E, F) UV melting curves of pG4-BS with one single nucleotide (**E**) or multiple nucleotide (**F**) bulge. (**G**) NMR spectra of selected pG4-BS with two bulges. (**H**) CD spectra of selected pG4-BS with two bulges.

All of the 32 predicted pG4-BS are comprised of >12 guanines and the bulges differ in size, location, and sequence composition. Most of the selected sequences are located in oncogenes or tumor suppressors associated with a wide range of cancer types (Table 2, Supplementary Table S8.1). First, we looked for evidence of G4-B formation utilizing NMR experiments. In our analyses, we consider three types of results as an indication of G4-B formation: a given pG4-BS is a positive hit if it possesses an NMR spectrum associated with either major G4, major + minor G4 or multiple G4 presence. Most sequences with single nucleotide bulges (e.g. BS13 (*RPN1* 1st intron), BS15 (*EGR1* promoter), and BS45 (*BLZF1* 1st exon)), displayed sharp G-tetrad associated NMR peaks (peaks in the region between 10.5 and 12.0 ppm on the NMR spectra), indicating the presence of a single major G4 conformation (Figure 8A). Other sequences (e.g. BS19 (*RGL2* promoter), BS26 (*NF1* 1st intron), and BS47 (*CBX4* promoter)), showed broad/weak G-tetrad proton peaks associated with the presence of major + minor G4 conformations. pG4-BS with multiple nucleotide bulges (e.g. BS24 (*RARA* 4th intron), BS31 (*ACTN* 1st intron)) also showed a type of broadened NMR peaks, suggesting the presence of multiple G4 conformations for these sequences (Figure 8B).

**Table 2.** Experimentally validated stable pG4-BS located in selected cancer-associated genes

Gene symbol	Entrez ID	Gene description	Chromosome	pG4-BS start	pG4-BS end	pG4-BS strand	Gene orientation	Sequence ID	Sequence with paired nucleotides on flanks	Number of intact G-stems	Number of bulges	Inserted nucleotides	Number of cytosines (C)	NMR spectrum model	CD Spectrum: antiparallel = 0, parallel = 1	UV melting temperature (°C)
*ACTN4*	81	actinin alpha 4	chr19	38648874	38648892	+	Sense	BS31	GA-GATCGG-T-GGG-A-GGG-A-GGG-TG	3	1	ATC	1	Single G4	1	51
*ANPEP*	290	alanyl aminopeptidase, membrane	chr15	89814896	89814915	-	Sense	BS36	TC-GGG-T-GGAG-TA-GTGG-TC-GGG-TC	2	2	A,T	1	Multiple G4	0	–
*BLZF1*	8548	basic leucine zipper nuclear factor 1	chr1	169368218	169368236	+	Sense	BS45	AT-GCGG-A-GGG-ACT-GGG-C-GGG-TC	3	1	C	2	Single G4	1	62
*CACNA2D2*	9254	calcium voltage-gated channel auxiliary subunit alpha2/delta 2	chr3	50503703	50503720	-	Sense	BS40	AA-GAGAG-A-GGG-A-GGG-A-GGG-AG	3	2	A,A	0	Single G4	–	<40
*CASP8*	841	caspase 8	chr2	201257751	201257767	+	Sense	BS20	GC-GGG-A-GGG-A-GGAG-A-GGG-CT	3	1	A	0	Major + minor G4	1	55
*CBX4*	8535	chromobox 4	chr17	79840014	79840034	+	Antisense	BS47	GC-GAGG-A-GGG-AAA-GGG-ACA-GGG-CG	3	1	A	0	Major + minor G4	0	38
*CCR7*	1236	C–C motif chemokine receptor 7	chr17	40563535	40563551	-	Sense	BS17	GT-GGG-T-GAGG-A-GGG-A-GGG-TT	3	1	A	0	Major + minor G4	1	63
*DNAJB1*	3337	DnaJ heat shock protein family (Hsp40) member B1	chr19	14518583	14518598	-	Sense	BS2	TT-GGG-C-GGAG-C-GGG-A-GGG	3	1	A	2	Major + minor G4	–	66
*E2F8*	79733	E2F transcription factor 8	chr11	19241765	19241782	-	Sense	BS33	TA-GAGG-C-GGG-A-GTGG-A-GGG-CG	2	2	A,T	0	Major + minor G4	1	50
*EGR1*	1958	early growth response 1	chr5	138464809	138464825	+	Sense	BS15	CT-GAGG-T-GGG-C-GGG-C-GGG-CC	3	1	A	2	Single G4	1	79
*EZR*	7430	ezrin	chr6	158818194	158818211	-	Sense	BS14	AC-GAGG-CA-GGG-C-GGG-C-GGG-CG	3	1	A	3	Single G4	1	72
*GDF15*	9518	growth differentiation factor 15	chr19	18387011	18387031	+	Sense	BS48	AT-GAGG-TCT-GGG-CT-GTGG-T-GGG-AC	2	2	A,T	1	Multiple G4	0	–
*LMO4*	8543	LIM domain only 4	chr1	87329129	87329146	-	Antisense	BS30	GA-GCTGG-A-GGG-A-GGG-A-GGG-AG	3	1	CT	1	Multiple G4	1	–
*MECOM*	2122	MDS1 and EVI1 complex locus	chr3	169321683	169321699	+	Antisense	BS16	TT-GTGG-A-GGG-A-GGG-T-GGG-AC	3	1	T	0	Single G4	1	76
*MECOM*	2122	MDS1 and EVI1 complex locus	chr3	169663029	169663045	-	Sense	BS28	AG-GGG-T-GGG-A-GGG-A-GGAG-AG	3	1	A	0	Multiple G4	1	–
*MYCN*	4613	MYCN proto-oncogene, bHLH transcription factor	chr2	15943554	15943572	-	Antisense	BS32	CT-GAATGG-A-GGG-A-GGG-A-GGG-TG	3	1	AAT	0	Single G4	1	43
*NF1*	4763	neurofibromin 1	chr17	31095380	31095397	+	Sense	BS25	CC-GTGG-C-GGG-C-GGG-A-GGTG-GG	2	2	T,T	2	multiple G4	1	–
*NF1*	4763	neurofibromin 1	chr17	31095380	31095399	+	Sense	BS26	CC-GTGG-C-GGG-C-GGG-AGGT-GGG-AG	3	1	T	2	Multiple G4	1	–
*NRP2*	8828	neuropilin 2	chr2	205685856	205685873	+	Sense	BS35	GA-GGG-C-GGAG-A-GAGG-C-GGG-AA	2	2	A,A	2	Single G4	1	49
*NTNG1*	22854	Netrin G1, transcript variant 11	ch1	107141271	107141287	-	Antisense	BS9	TT-GGG-C-GGAG-C-GGG-AG-GGG	3	1	A	2	Major + minor G4	1	–
*PTGFRN*	5738	prostaglandin F2 receptor inhibitor	chr1	116909887	116909902	+	Sense	BS1	TT-GGG-A-GGCG-C-GGG-C-GGG	3	1	C	3	Major + minor G4	–	69
*PVT1*	5820	Pvt1 oncogene	chr8	127795677	127795693	+	Sense	BS51	GT-GTGG-T-GGG-T-GGG-C-GGG-GG	3	1	T	1	Major + minor G4	–	>80
*RARA*	5914	retinoic acid receptor alpha	chr17	40341607	40341624	+	Sense	BS18	AC-GGG-CA-GCGG-T-GGG-T-GGG-TC	3	1	C	2	Single G4	1	70
*RARA*	5914	retinoic acid receptor alpha	chr17	40350488	40350505	+	Sense	BS24	CT-GATGG-A-GGG-A-GGG-A-GGG-AA	3	1	AT	0	Major + minor G4	1	47
*RASSF1*	11186	Ras association domain family member 1	chr3	50337643	50337660	+	Antisense	BS22	GA-GGG-C-GGG-AA-GGTG-C-GGG-AA	3	1	T	2	Single G4	1	63
*RGL2*	5863	ral guanine nucleotide dissociation stimulator like 2	chr6	33299346	33299364	-	Sense	BS19	GT-GGG-TT-GTGG-TA-GGG-A-GGG-TA	3	1	T	0	Major + minor G4	0	61
*RPN1*	6484	ribophorin I	chr3	128650848	128650864	-	Sense	BS13	CA-GTGG-C-GGG-A-GGG-C-GGG-CT	3	1	T	2	Single G4	1	78
*VWF*	7450	von Willebrand factor	chr12	6124638	6124656	-	Sense	BS46	TT-GTGG-T-GGG-AAA-GGG-A-GGG-TG	3	1	T	0	Major + minor G4	1	56

A few other sequences (e.g. BS19 (*RGL2* promoter), BS26 (*NF1* 1st intron), and BS47 (*CBX4* promoter)), showed broad/weak G-tetrad proton peaks. The lack of well-defined signal peaks suggests the absence of a stable G4 conformation and/or the presence of alternative structures. Such structures (weak form G4, duplex DNA + G4 or only duplex DNA) were considered as ‘non-G4’ structures.

We note that the presence of G4 imino peaks does not fully guarantee that stable G4-B formation takes place. For this reason, we complemented our NMR studies with CD spectroscopy (Figure 8C, D) and UV melting curve analysis (Figure 8E, F) on selected sequences. In CD spectroscopy experiments, parallel G4s usually show a positive peak around 260 nm and a negative peak around 240 nm. Most of the pG4-BS with single nucleotide bulges showed evidence of forming parallel G4 structures, while a few did not (e.g. BS19 (*RGL2* promoter), BS47 (*CBX4* promoter)) (Figure 8C). These observations support our previous NMR results, indicating the lack of stable G4 formation in these sequences. We also observed that the incorporation of multiple nucleotide bulges did not diminish the ability of pG4-BS to form G4s (Figure 8D).

It has been previously shown that bulges may decrease the stability of G4s with right-handed conformations (26). To assess the impact of bulges on stability, we plotted the fraction of folded DNA obtained from UV absorption at 295 nm as a function of the temperature. Melting temperature is defined as the temperature at which 50% of the total DNA remains folded. We observed a wide range of melting temperatures for pG4-BS with either single (Figure 8E) or multiple (Figure 8F) nucleotide bulges. This result supports our previous hypothesis that differences in sequence composition can give rise to G4s that are highly diverse in both topology and stability.

In separate experiments, we validated sequences that contain two bulges. The NMR spectra of these pG4-BS indicate the presence of multiple G4 conformations (Figure 8g). Certain sequences (e.g. BS35 (*NRP2* 1st intron) indicated the formation of a single major G4 structure, while others (e.g. BS48 (*GDF15* 1st intron)) may instead adopt several different conformations depending on their environment. The CD spectra revealed that despite the higher number of interruptions, most of the tested sequences were able to form parallel G4s, similar to pG4-BS with a single bulge (Figure 8H). The UV melting curves of selected pG4-BS with two bulges revealed that the G4s formed by these sequences possess stability comparable to G4s with a single multiple nucleotide bulge (Supplementary Figure S9). However, the thermal stability of pG4-BS with one single nucleotide bulge was on average higher. All of the sequences tested in our study, along with their genomic coordinates and the genes and specific gene segments they are associated with are available in Supplementary Table S8.

### Molecular characterization of *E2F8* promoter-associated pG4-BS

Recent work has shown that promoter G4s are associated with high transcription levels in open chromatin (76). However, other groups have reported that promoter G4 formation is not dependent on transcriptional activity, but rather the chromatin structure (77). Due to genome instability, mutations, and signal perturbation, G4s in key regulatory regions of cancer-associated genes could modulate gene expression and have downstream effects linked to the dysregulation of gene expression and uncontrolled cell proliferation. We hypothesize that G4-BS in promoter regions of genes encoding transcription factors (TFs) are essential regulatory signals for transcription processes in cells. The hypothesis can be supported by the pG4-BS predicted by our method in the proximal promoter site of the pro-oncogenic transcription factor *E2F8*. E2F8 is part of the E2F family of transcription factors and is a key regulator of proper cell-cycle progression (78). Importantly, the overexpression of E2F8 has been shown to induce tumorigenesis and progression while its forced downregulation resulted in the inhibition of tumorigenic phenotypes in several human cancers, such as hepatocellular carcinoma (79), cervical cancer (80), lung cancer (81) and colon cancer (79–82). Taken together, these results suggest that promoter G4 formation precludes unique active gene transcription regulation and in the context of other signals may play an important role in the stabilization (or destabilization) of permissive chromatin.

**Figure 9.**
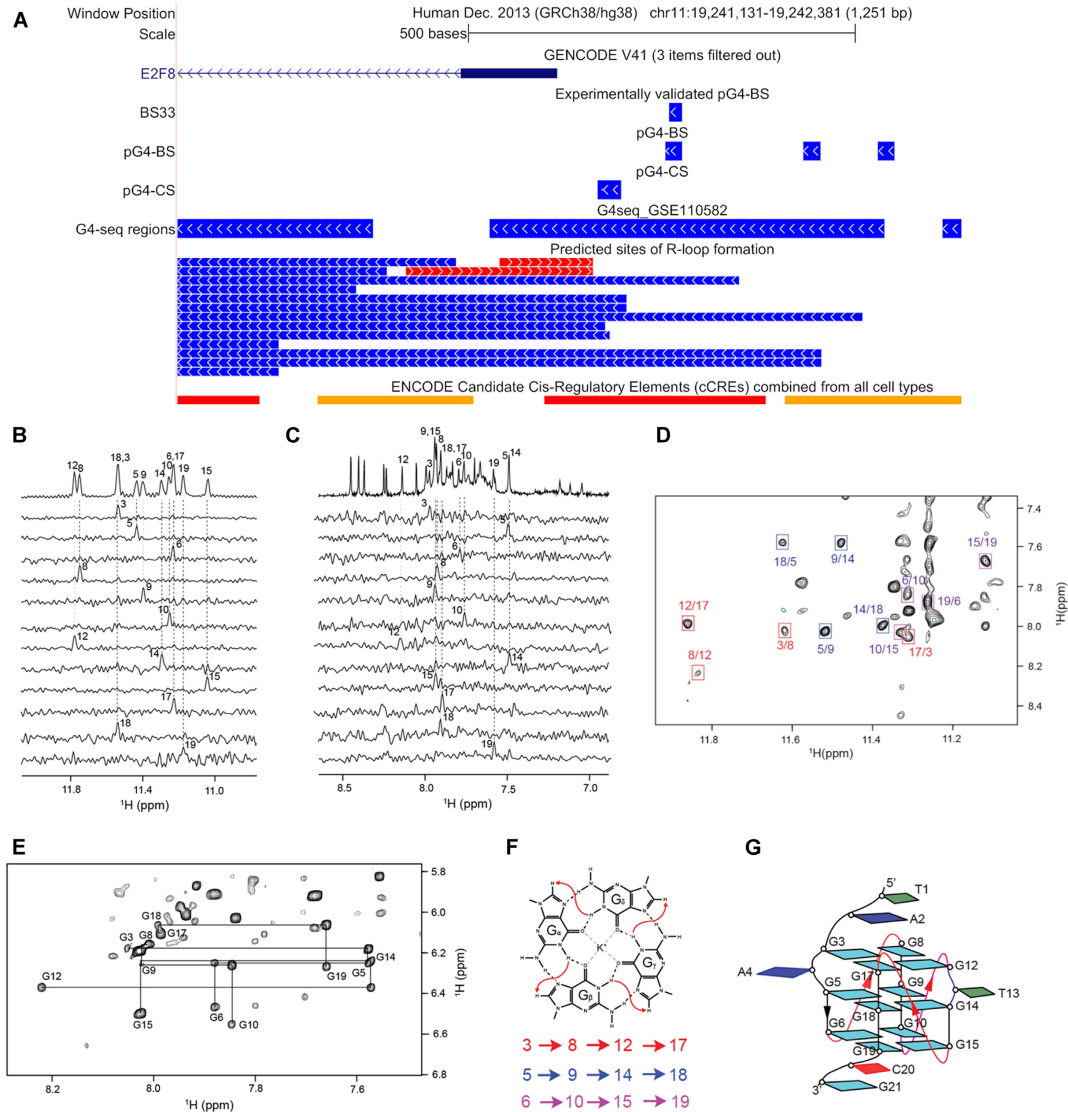
(**A**) BS33 is co-localized with sites of RLFS, CpG islands and ENCODE cCREs. (B, C) NMR spectral assignments of (**B**) guanine imino-protons and (**C**) guanine H8-protons using site-specific 2% 15N-labeled samples. (**D**) NOESY spectrum showing the imino-H8 connectivities around different G-tetrads. The characteristic guanine imino-H8 cross-peaks for G-tetrads are framed and labeled with the imino proton assignment in the first position and that of the H8 proton in the second position. Guanines in different G-tetrads are indicated by different colors. (**E**) Specific imino-H8 connectivity pattern around a G-tetrad is shown together with the distribution of guanines in each G-tetrad. (**F**) NOESY spectrum showing H8/H6-H1′ NOE sequential connectivities of BS33. Intraresidue cross-peaks are labeled with the corresponding residue numbers. (**G**) Schematic structure of the G-quadruplex adopted by BS33.

We have identified two pG4-BS sequences located in the proximal promoter region of the *E2F8* gene (Figure 9A), both of which belong to the G2B2 model. These sequences are located ∼150 nucleotides upstream of TSS of *E2F8*. These pG4-BS are co-localized with experimentally-defined G4 signals (G4-seq (14,51,75), RLFS clusters (14,51,75), CpG islands, and ENCODE promoter-like elements (TF binding sites). None of the shown pG4-BS share any G-stems with nearby G4-CS, thereby representing a potential unique regulatory G4 structure in the *E2F8* promoter. In addition, both pG4-BS are further classified as ‘regulatory’ pG4-BS, due to direct overlapping with transcription factor binding sites and/or DNAse I hypersensitive regions (Figure 9A). Note that we also observed pG4-CS in the proximity of the *E2F8* TSS (Figure 9A). However, the broader gene regulatory signal landscape, including TF binding sites and RLFS composition on both DNA strands, is not the same for these pG4-CS as for the studied pG4-BS.

For a deeper understanding of G4-B conformations, we performed the in-depth structural characterization of the *E2F8* promoter sequence BS33 (d[*TA*-**G**A**GG**-C-**GGG**-A-**G**T**GG**-A-**GGG**-*CG*], tetrad guanine residues in bold, bulging nucleotides underlined, and flanking sequences in italic). This pG4-BS belongs to the G2B2 model. BS33 is located on the non-template strand and is 162 nucleotides upstream of the *E2F8* TSS. Furthermore, BS33 is co-localized with experimental G4 signals, RLFS, CpG islands and ENCODE promoter-like elements (Figure 9A). Additionally, BS33 does not share any of its G-clusters with pG4-CS, thereby representing a potential unique regulatory G4 structure in the E2F8 promoter. Imino and aromatic proton resonances of BS33 were unambiguously assigned using a site-specific low-enrichment approach (Figure 9B, C) with 2% 15N-labeled samples. The folding topology of BS33 was determined from the identities of each G-tetrad. The arrangement of guanines in the G-tetrads was determined using the H1-H8 cyclic connectivity pattern from the NOESY spectra (Figure 9d). We observed NOE cross-peaks (in red squares) between G3(H1) and G8(H8), G8(H1) and G12(H8), G12(H1) and G17(H8), and G17(H1) and G3(H8). This suggests the formation of the (G3.G8.G12.G17) tetrad. In the same way, cross-peaks in the purple and blue squares indicate the arrangement of the (G5.G9.G14.G18) and the (G6.G10.G15.G19) tetrads respectively (Figure 9E). Moderate intensities observed between H8 and H1 of all the guanines (Figure 9F) suggest an anti-glycosidic conformation, consistent with a parallel G4 structure. To further support the folding topology, we performed a solvent exchange experiment. When dissolving the sample in D2O solution, imino protons of guanines belonging to the two outer G-tetrads disappeared quickly, while those belonging to the inner G-tetrad remained protected. The linewidths of guanine amino proton peaks also indicated that G5, G9 and G14 were situated in the middle tetrad, supporting the proposed folding topology (data not shown). Taken together with the characteristic CD spectra of BS33, these results indicate the formation of a three-layer parallel G4-B with two bulges, A4 and T13 (Figure 9G).

### Accuracy, specificity, and sensitivity of stable pG4-BS predictions

To quantify the accuracy of our pG4-BS models, we considered the results of our experimental validation as well as data collected from our previous studies that includes 33 potential G4-forming DNA oligomers (Supplementary Table S8.1). The G4-forming ability of these sequences was assessed using the same structural stability assays as the ones we utilized in the current study. The oligomers in this dataset include 26 sequences corresponding to one of our pG4-BS models, out of which 25 showed evidence of stable G4 formation, and hence were considered as ‘true-positive’. Out of the remaining sequences, 6 oligomers match alternative pG4-BS compositions (G0B4 or G1B3) and did not form stable G4 conformations. We considered these six non-G4-forming sequences as negative controls. The final oligomer in this dataset is a representative stable canonical G4-forming sequence that we considered as a positive control. By combining this dataset of artificial sequences (*n* = 32) with our previous set of experimentally tested pG4-BS (both artificial and human genome-derived; *n* = 50) our total collection includes 82 samples (Supplementary Table S8.1–2). The number of experiments and our experimental design of the collected data allowed for the estimation of the sensitivity, specificity, accuracy, and positive likelihood score of our G4-BS models. As above, experimental G4 formation was considered ‘positive’ if the tested sequence showed evidence of possessing a single major, major + minor, or multiple G4 conformations. Other instances (weak form G4, duplex DNA + G4, two-layer G4, duplex DNA) were considered as ‘negative’ cases of experimentally detected G4 formation. Using this criterion, we categorized the tested sequences as either ‘true positive’ (predicted and experimentally detected G4; *n* = 56), ‘true negative’ (no predicted or experimentally detected G4; n = 19), ‘false positive’ (predicted, but no experimentally detected G4; *n* = 7) or ‘false negative’ (no predicted, but experimentally detected G4; n = 0). Using exact categorical analysis (Cytel Studio), we found that the accuracy, specificity, sensitivity, and positive likelihood score of G4-BS models are 91.5%, 73.0%, 100% and 3.71, respectively. The weighted Kappa correlation coefficient value was 0.78 (95% CI [0.702, 0.858]); two-sided *P* = 1.35E-12).

## DISCUSSION

### pG4-BS in the human genome

In this study, we identified a large subfamily of G-rich sequences in the human genome that contain interrupted stems and are likely to possess stable G4-B forms. Conventional G4-S predicting algorithms (most commonly based on the *G_3+_N_1-7_G_3+_N_1-7_G_3+_N_1-7_G_3+_* pattern) overlook these sequences. Importantly, while more lenient bioinformatics algorithms exist today (38), they are still constrained by the mostly unknown parameters required for productive G4 formation by these ‘imperfect’ sequences. Some newer G4 mining algorithms, for example, G4Hunter (39) and ImGQfinder (26), include options to approximate the G4-B forming ability of query sequences. One of the main problems with G4-B prediction is that the intrinsic G4 forming ability can vary based on the degree of G-stem alterations and their positions in the sequences. Without knowledge of the sequence constraints for G4-B, the biological interpretation of computational predictions is dubious and makes genome-wide mapping of potentially functional G4-BS difficult.

The construction of the G4-BS models is based on the results of our NMR, CD and thermodynamic stability assays. Using these results, we established three stable G4-BS models that allow for varying degrees of sequence alterations without compromising the thermodynamic and structural characteristics of these sequences. The results showed that the bulges can be formed within G4 under different conditions; however, our G4-BS models only select sequences with certain nucleotide compositions and positions of non-guanine nucleotides, predicting stable G4 conformations. While the sequences tested in this study can form stable G4 structures at an accuracy of 92%, all of them defy the description of the traditional G4 motif (*G_3+_N_1-7_G_3+_N_1-7_G_3+_N_1-7_G_3+_*), currently used in most bioinformatics searches and experimental studies for identifying potential G4FS in the genomes.

Ultimately, we selected three G4-BS models that are most likely to encompass pG4-BS possessing the stability required for G4-B formation and, as an extension, G4-related biological and pathobiological functions. We further implemented filtering steps to remove sequences with contiguous **C** bases to reduce the number of false-positive results. The logic that the presence of C clusters can be detrimental has already been used in other G4 mining tools. For example, G4Hunter is a newer G4-finding mining tool with a scoring system that takes into account the GC-skewness of the DNA sequence (39). Specifically, the algorithm scores the individual nucleotides based on their expected effect on the overall thermodynamic stability of the G4 structure. In this scoring schema, contiguous cytosines receive lower scores concerning the number of **C** bases in the given cluster. Additionally, some DNA sequences with frequent C insertions may prefer stem-loop/hairpin conformations due to the thermodynamic advantage such sequence compositions provide. In line with this, we removed pG4-BS with contiguous **C** runs (e.g. **CC**, **CCC**), thereby filtering for sequences with more favorable G4 forming ability.

Our search algorithm identified 1935686 pG4-BS, over 60% (1 169 397) of which do not overlap with any pG4-CS. These unique pG4-BS represent a population of alternative G4-S that could possess novel structural elements and DNA conformations with multiple gene regulatory functions. We observed differences in the population sizes of pG4-BS belonging to each of the three G4-BS models. These differences in the size of different pG4-BS populations could be explained by the variations in the underlying sequence. We expect sequences belonging to the G2B2 model to be generally less stable compared to G3B1, due to the presence of two bulges. Due to this reduced stability, G2B2 type pG4-BS could potentially be involved in molecular processes requiring the transient presence of G4s. At the same time, the reduced stability could explain the abundance of these sequences in the genome, as they are less likely to form unscheduled G4s (compared to G3B1 type pG4-BS).

We note that while our models allow for the selection of pG4-BS that likely possess the ability to form stable G4-B, not all identified pG4-BS can do so. Additionally, there likely are sequences that can form stable G4-B, but that do not adhere to any of our models. One such example is the G4-B formed by the *TP3* DNA sequence (83). *TP3* forms a three-layered, intramolecular, and thermodynamically stable G4-B, and is located in the promoter of the *PARP1* gene, 125 nucleotides upstream of the TSS. Based on the configuration of its stems and loops, this sequence would belong to the G3B1 model, with a single adenine (A) inserted into the second stem. The reason it is excluded from our analysis is due to the presence of contiguous **C** bases in its sequence. The results of thermodynamic stability assays of *TP3* show that some of the excluded sequences may indeed be capable of G4 formation. Nevertheless, based on our analysis of the FD of the occurrences of pG4-BS, we expect our method to capture the majority of true positive pG4-BS within the human genome. Based on the total length of merged pG4-BS regions, we observed that pG4-BS regions cover in total 0.26% (3 494 639 bp) of the non-template strands of human protein coding gene bodies and promoters. This number is more than three times the coverage observed for pG4-CS regions (0.0084%; 1 137 024 bp) in the same context.

One of the key requirements for NGS methods is adequate accuracy to map sequences of interest to the target genome and identify their boundaries (54,84). Our computational simulation and comparison of the boundaries of pG4-CS and pG4-BS showed that computational prediction provides essentially higher resolution for genome-wide sequence boundary mapping of putative sites of G4 formation regions than the G4-seq method. However, experimental approaches can highlight biological differences that cannot be measured using purely computational methods. In Figure 5a, we present a genome browser image showing that our computationally predicted pG4-BS are embedded in the signal peak boundaries detected via the G4-seq method. In addition, we showed that the GC%, an essential characteristic of G4-S, is significantly different between G4-S and G4-seq regions (K–S test, *P*-value < 2.2e–16) (Supplementary Figure S6c).

We also utilized G4 CUT&Tag datasets containing *in vivo* detected sites of G4 formation in multiple cell lines. While the differences in sequence lengths between CUT&Tag peaks and pG4-BS and the lack of replicate experimental samples made cell line-specific comparison of pG4-BS profiles unfeasible (Supplementary Figure S6a and Supplementary Table S7.1), we carried out the analysis of a ‘pooled’ datasets. This contained the peak regions pooled together from the available six DNA samples (for further details, see Methods). Our results have shown that pG4-BS are co-localized with 34% (15 703 out of 45 968) of peak regions in the ‘pooled’ G4 CUT&Tag dataset (Figure 6c and Supplementary Table S7.3). We observed similar trends in the number of pG4-CS supported peak regions in the ‘pooled’ G4 CUT&Tag datasets (16% supported) and for G4-seq supported regions (47%). We have also shown that pG4-BS are similarly enriched as pG4-CS in G4 CUT&Tag-derived peak regions compared to background sequences (Supplementary Table S7.3). Taken together, we conclude that pG4-BS supports a large percentage of *in vivo* detected G4 sites.

During our comparison between datasets from our computational approach, G4-seq and G4 CUT&Tag we observed advantages and disadvantages for each of these methods. Computational predictions were the most accurate in determining G4-forming sequence boundaries, but such data cannot give reliable information about the underlying biological functions. The G4-seq method has worse resolution and is also unable to account for biological context, however, the method itself does not utilize models to identify potential G4 forming sequences and discriminate G4-CS versus G4-BS. This allows G4-seq to escape some of the limitations inherent to any model. Finally, G4-BS and G4-CS embedded in the CUT&Tag peak regions could, however, filter specific regions and expand the significance and biological context of experimental data, making them highly valuable for discovering G4s-specific therapeutic targets.

In summary, our pG4-BS models highlight a finer nucleotide density structure at key gene regulatory regions, including putative promoters, exon-intron junction sites, TSS and TTS. Thereby, our data-driven computational prediction method provides higher resolution identification of potential sites of G4-formation genome-wide compared to purely experimental NGS-based approaches. To improve the resolution of genome-wide experimental G4 detection methods, pG4-BS could be used as quality control data. Our high-resolution G4-BS mapping could also be used for a deeper, structure-based classification of potential G4-forming sites and specific genome engineering and/or therapeutic targeting. Utilizing a combined experimental validation dataset of sequences that were either predicted to form G4-B or not, we calculated an accuracy of 91.5% for our pG4-BS.

### Biological implications of pG4-BS

G4-B could form under the same conditions as G4-C, chiefly in single-stranded DNA. The dissociation of DNA strands most commonly occurs during transcription and replication. In these biological processes, the DNA strands can persist as single strands in the genomic regions encompassing pG4-BS long enough for G4-B formation to take place.

We analyzed the distributions of pG4-BS across the promoters and gene bodies of protein-coding genes in the human genome. Out of the 478263 non-repeat overlapping pG4-BS regions, 285994 regions are spread across 17 993 protein-coding genes. We observed that the proportion of pG4-BS-positive genes was >2 times the proportion of pG4-CS-positive genes in distinct gene segments, such as the 5′ and 3′ UTRs, exons and promoter regions. These results support our hypothesis that pG4-BS sequences show potentially stronger gene regulatory signals compared to pG4-CS.

The EFDs of pG4-BS regions across the TSS and other critical gene segments showed strong similarity with the EFD of pG4-CS. However, we also saw strand-specific differences in the FDs that indicate that pG4-BS may possess functions different compared to those of pG4-CS. Further analysis focusing on the different pG4-BS models revealed that, after normalization, the three models showed similar distribution patterns. Differences were mainly observed at sites of major pG4-BS presence, where the less common models (e.g. G3B1 and G3B2) showed stronger enrichment than the most common G2B2 model. This suggests that sequences belonging to different models may form G4-B that could possess unique functions depending on their 3D structure. Many of the identified pG4-BS are located in biologically important regulatory loci. Out of the 20 715 pG4-BS regions mapping to the promoter regions of protein-coding genes over 89% intersected with chromatin accessibility regions and/or transcription factor binding sites. Taken together with the observation that pG4-BS is similarly strongly enriched in the TSS-proximal regions as pG4-CS; this suggests that such pG4-BS could exert regulatory functions in both normal and pathological conditions.

The distribution of the number of pG4-BS regions per gene assumes the pattern of a skewed distribution, where a few observations are especially common, and most others are rare. Such distribution functions can be approximated based on the K–W model (69,70) (Supplementary Methods). Based on the EFD, we observed that our pG4-BS finding algorithm captures the majority of potential pG4-BS sequences defined by our models in the human genome.

In our GO analyses, we observed several highly significant enriched terms between gene sets containing the genes with regulatory pG4-BS or pG4-CS. These terms included GO categories related to development, transcriptional regulation, and the nervous system. Such categories were previously reported for pG4-CS and duplex stem–loop containing G4s (42,45). Interestingly, the strongest enrichment in terms was observed for the subset of genes that contained *both* regulatory pG4-BS and regulatory pG4-CS in their *TSS-proximal region*. This categorization suggests that there could be positive micro-evolutionary pressure on such genes to keep both types of G4-S in their promoter regions. Furthermore, we saw that genes with regulatory pG4-BS located in the TSS-proximal region on both non-template and template strands (referred to as bf-pG4-BS) were highly enriched for the previously observed terms, while genes with pG4-BS regions located only on either strand were not. These findings suggest that the combined presence of pG4-BS on both strands may be required for the effective regulation of certain genes. Similar findings were observed for genes containing regulatory pG4-CS in their promoters: genes with bf-pG4-CS (e.g. pG4-CS present on both strands) were enriched for biological terms, while those with pG4-CS on a single strand only were not. In a follow-up analysis, we saw that regulatory G4-S presence on both strands was associated with biological and functional term enrichment. This indicates that G4-C and G4-B could work in tandem and constitute hybrid bf-G4-S architectures. Finally, we found that genes with both types of bf-G4-S are enriched with GO terms over genes with only one type of bf-G4-S pair. Taken together, our findings suggest that G4-S likely fulfill at least some of their biological functions not as single entities, but as part of a functional unit, that spans both DNA strands. Furthermore, the presence of both bf-pG4-BS and bf-pG4-CS may be necessary for specific G4-mediated biological functions. The exact reasons for this are not yet clear. One possible explanation is that the simultaneous presence of pairs of different types of pG4-S on both strands can enable the more profound stabilization of open chromatin and recruitment of transcription factors. While we did not observe a strong divergence between the distributions and localizations of pG4-BS and pG4-CS in our analyses, these findings do indicate that G4-S specificity may be essential in some contexts. Specifically, genes with only bf-pG4-CS did not show significant GO term enrichment, while we did observe GO term enrichment for gene sets with only bf-pG4-BS. The combined presence of different G4-S on both strands could be essential for fine-tuning the expression of certain genes by having multiple levels of regulatory G4 signals in place.

In the pathway enrichment analysis of the 2761 regulatory bf-pG4-BS(+) protein-coding genes, we saw the enrichment of multiple signaling pathways. This included pathways (e.g. Wnt signaling pathway, PI3K-Akt signaling pathway, Notch signaling pathway) that are on one hand crucial regulators of cellular homeostasis, survival and growth, and on the other are potential drivers of disease (e.g. different types of cancer, neurodegenerative disease, cardiovascular disease). Furthermore, many of the bf-pG4-BS-containing genes are associated with more than one signaling pathway. This overlap between pathway members could cause the transcriptional dysregulation of a single gene to affect multiple signaling pathways. The ability of G4s to regulate transcription has been well documented in recent publications (76,77,85). Our findings of bf-pG4-BS located in the promoters of such ‘hub’ genes, combined with extensive reports about the role of G4s in transcriptional regulation highlight the need for further studies to elucidate the functions of bf-pG4-BS in the human genome.

Additionally, our results suggest essential structural and regulatory roles for pG4-BS in the biological and pathobiological functions of a set of nervous system-associated genes. The genes *Shank1* and *Shank2* (containing 74 and 394 pG4-BS regions, respectively) encode proteins that are members of the Shank family of proteins functioning as molecular scaffolds in excitatory synapses, and whose deletion was associated with increased susceptibility to autism spectrum disorder (86,87). *Kirrel3* (274 associated pG4-BS regions) is a member of the transmembrane immunoglobulin superfamily and is expressed in the fetal and adult brain and kidney podocytes. Mutations of *Kirrel3* have been associated with intellectual disability and autism (88). *SORCS2* is another gene highly populated by pG4-BS regions (410 associated pG4-BS regions), whose mutations may carry the increased risk of reduced neuronal synaptic plasticity, bipolar disorder, attention deficit-hyperactivity disorder and schizophrenia (89). In addition, the most pG4-BS populated human gene is *PTPRN2* (contains 669 pG4-BS regions), a protein tyrosine phosphatase previously shown to contain a large number of non-canonical G4-S of a different type (45). PTPRN2 holds critical functions in vesicle-mediated secretory processes, such as insulin secretion and the accumulation and secretion of neurotransmitters (e.g. norepinephrine, dopamine) and pituitary hormone in females (e.g. luteinizing hormone (LH), follicle-stimulating hormone (FSH)). Furthermore, altered PTPRN2 expression has been connected to breast cancer cell migration via cytoskeleton remodeling (90).

Importantly, all the above-mentioned genes are strongly associated with RLFS clusters. Specifically, *PTPRN2* and *SORCS2* are two of the most RLFS-rich gene in the human genome, containing 140 and 115 RLFS, respectively. Sites of R-loop formation are often associated with increased cell stress and genome instability, due to DNA breaks, potentially promoting the development of cancer and other diseases (91). According to the COSMIC database, *PRPRN2* and *SORCS2* point mutations were detected in ∼18% and ∼13% of breast cancer samples, and ∼26% and ∼16% of prostate cancer samples, respectively (92). There is mounting evidence that G4s, including G4-B, and R-loops can form functional units, influencing the formation and stability of both of these structures (59,93).

It has been postulated that G4-R-loops, R-loops with at least one G4 formed in the displaced DNA strand, could have increased stability and potentially unique functions (1). As such, pG4-BS co-localized with RLFS may act in concert, potentially promoting R-loop formation and/or stabilization. We found that among 229000 merged RLFS clusters identified in humans over 50% overlap with pG4-BS, pG4-CS or both (Figure 7a). However, uniquely pG4-BS(+) RLFS were three times as common as RLFS clusters with only pG4-CS support. We also observed a significant strand-specific association between G4-S and RLFS, mostly near the initiation and termination regions of RLFS clusters. Roles of the strand-specific pG4-BS and pG4-CS at the 5′-end 3′-end of RLFS require further investigation.

G4 stabilizing ligands are increasingly considered as potential therapeutic agents for the treatment of cancers. One of the main challenges of targeting G4s is avoiding unwanted side effects, due to the low specificity of these compounds (28). G4-B represents a subset of G4-like structures with highly diverse topologies. In turn, the different topologies could allow for the future development of G4 targeting ligands with higher specificity. Importantly, the screening of ligand binding over different subgroups of G4-B could represent a way to reduce off-target G4 stabilization, allowing for the discovery and selection of ligands with higher specificity and less severe side effects.

The ligands that bind G4-conformations can have either a positive or a negative impact on R-loop formation and stability (60,94). For instance, a recent study showed that the porphyrin metabolite hemin, which primarily binds parallel G4s, provides partial reduction of RNA-DNA hybrid signal genome-wide in cells (94). We suggest that computational predictions for bulged G4s and R-loops can select higher-confidence G4s and R-loop signals from NGS-based methods (e.g. G4 CUT&Tag, DRIP-seq). This approach could offer new strategies for investigating G4-binding ligands' effects on R-loop initiation and stability.

### G4-B structures of pG4-BS

Accurate genome-wide prediction and identification of pG4-BS will further our understanding of the biological functions of these structures and broaden our definitions regarding G4s. To validate our findings, we selected a representative sample of pG4-BS from clinically relevant genes and carried out the structural characterization of these sequences using a combination of NMR, CD spectroscopy and UV melting experiments. The selected pG4-BS include sequences from various gene segments of cancer-associated genes, such as *EZR*, *PRN1*, *RARA* and *NF1*. Among the tested sequences, we saw evidence of diverse G4-B topologies. While some sequences showed a single dominant G4-B (e.g. BS13:*RPN1*), others either showed structural polymorphism (e.g. BS20:*CASP8*) or failed to form stable G4-B (e.g. BS42:*UBE2D3*).

Our results suggest that the topology and stability of G4s formed by pG4-BS is highly diverse and is physically dependent on the underlying nucleotide's sequence composition. We hypothesize that the sequence composition of the loops connecting G-stems, and the nucleotide composition and location of the bulges all play a role in determining the G4-B-forming ability of these sequences. Generally, pG4-BS with fewer bulges (including both the number and length of bulges) tend to be more stable. However, even pG4-BS with a strong deviation from the G4-C motif can form stable G4-B, and thereby potentially fulfill biological functions. The highly diverse structural landscape of G4-B may allow for more varied roles compared to G4-C.

Additionally, we validated the formation of G4-B by three pG4-BS sequences associated with the 1st intron of *NF1* (BS25, BS26 and BS27). NF1 is a tumor suppressor, which was observed to be inactivated through loss-of-function mutations in mouse mammary tumors and human BCs (95). Both BS25 and BS26 are located in the 1st intron of *NF1*, however, BS25 contains two bulges, while BS26 contains only one bulge. Based on our results, neither of these sequences possess a single dominant G4-B conformation and likely alternate between multiple conformations. Previously, we have shown that *NF1* contains a high number of transposon insertion sites, potentially increasing the rate of mutation at the locus (96). BS27 is a variant of the BS26 sequence that models the potential effects of mutations on the ability of pG4-BS to form G4-B. The association between non-B DNA, specifically G4s, and variation in nucleotide substitution frequencies that can contribute to genetic diseases has been reported (16,97,98). Importantly, Guiblet et al have shown that G4s sites are associated with higher mutation rates and that nucleotide substitutions occur in loops more frequently compared to stems (16). BS27 contains a modified third loop: **ATTT**, instead of the **AGGT** found in BS26, modeling the potential effects of nucleotide substitutions in the loops of pG4-BS and changes in G4-B forming ability. The modification of this single site causes BS27 to adopt a single dominant G4 structure (Figure 8A). Based on our other results, BS27 likely adapts a parallel G4 conformation, and possesses a melting temperature of 67°C, suggesting high thermodynamic stability. These results highlight that mutations in the DNA sequence could promote the formation of highly stable G4-B. In turn, persistent unscheduled G4 presence may influence the regulation and expression of associated genes, thereby contributing to disease initiation and progression. We note that both BS26 and BS27 contain a loop of four nucleotides, exceeding the limits set by our pG4-BS identifying algorithm. This suggests that pG4-BS with more complex sequence variation could exist, and these may possess distinct features and biological functions. Based on these results, we can potentially modify our search criteria in the future and expand the types of captured pG4-BS. We also performed the detailed structural characterization of BS33, which is a pG4-BS located in the promoter region of the *E2F8* gene. Our results indicated the formation of a three-layered parallel G4-B structure containing two bulges. The potential biological relevance of this G4-B is supported by the proximity of experimental G4 signals, RLFS and different gene regulatory elements.

In summary, we have, for the first time, modeled and validated DNA sequences that form stable G4-like structures *in vitro*, called G4-B. In the human genome, we discovered a large family of G4-BS that is distinct from canonical G4s and found that G4-BS members are present in 92% of human protein-coding genes or their proximal vicinity. G4-B structures formed by pG4-BS that were identified in a representative subset of cancer-associated genes were experimentally verified. We determined the regulatory signal landscape of pG4-BS. Our experiments and modeling suggest that G4-BS form stable G4-B conformation(s) in the promoters of genes associated with transcriptional regulation, cancers and the nervous system. Finally, pG4-BS located in the proximal promoter of the *E2F8* gene was experimentally validated by reconstructing a detailed folding topology of a G4-B with two G-stem bulges. pG4-BS forms novel and stable G4 structures, expanding the definition of physiologically and pathologically relevant G4-associated regulatory signals, and potentially revealing future therapeutic targets and regulatory molecules.

## SHORT SUMMARY

1. Three experimentally validated bulged G4 sequence models for the identification of stable G4-B genome-wide are introduced.
2. 478263 merged pG4-BS were preferentially identified in the transcription regulatory sites of 90% of human protein-coding genes.
3. Over 2800 genes with pG4-BS on both strands in the gene promoter region are overrepresented by the genes essential for transcription regulation, cell differentiation, ion channels, genome instability, immune response, cancer and neurodegenerative diseases.
4. G4-B formation by a representative subset of pG4-BS associated with the human cancer genes located in the promoter or gene body regions was experimentally validated.
5. G4-B conformations predicted by our G4-S models are highly enriched in independent G4-seq and G4 CUT&Tag datasets.
6. We predicted and reconstructed the 3D conformation of a stable G4-B in the *E2F8* promoter.

## DATA AVAILABILITY

Raw pG4-BS data is available in the Mendeley Data database under DOI: 10.17632/w37rx9hpb7.1. The processed datasets, including the coordinates of merged pG4-BS regions, merged pG4-CS regions and merged pG4-BS model-specific regions are available at the following link: https://doi.org/10.6084/m9.figshare.22110965. A UCSC genome browser session containing the tracks used in this study, including computational and experimental data is available (https://genome.ucsc.edu/s/Csaba_Papp/G4BS_project_Hg38). The track hub is also available via the URL https://github.com/pappc/g4_hg38/blob/main/bigBedFiles/trackDb.txt (archived version is available at: https://doi.org/10.6084/m9.figshare.22144427). Additional data from this study is also available in the supporting materials.

## Supplementary Material

gkad252_Supplemental_Files
