# Supplementary material for: Stable bulged G-quadruplexes in the human genome: identification, experimental validation and functionalization
Source: Nucleic Acids Res. 2023 Apr 24;51(9):4148–77. doi: 10.1093/nar/gkad252 (PMC10201450; doi:10.1093/nar/gkad252)
Supplement: gkad252_Supplemental_Files [file gkad252_supplemental_files.zip › Supplementary files_CP4_VK2.final.docx]

# Supplementary files

## 1. Supporting Materials and Methods

### Reagents

ABI 394 DNA/RNA synthesizer: Thermo Fisher Scientific
Bruker 600 and 700 MHz spectrometers: Bruker
JASCO V-650 spectrophotometer: Jasco
JASCO-815 spectropolarimeter: Jasco

### Web sites/Databases/Software

biomaRt R package: <https://bioconductor.org/packages/release/bioc/html/biomaRt.html>
DAVID bioinformatics resources (2021): https://david.ncifcrf.gov/home.jsp
Enrichr: <https://maayanlab.cloud/Enrichr/>
ENSEMBL 105: <http://dec2021.archive.ensembl.org/index.html>
FELIX: <http://www.felixnmr.com/>
SkeweDF R package: <https://cran.r-project.org/web/packages/SkeweDF/index.html>
UCSC Genome Browser RepeatMasker track: <https://genome.ucsc.edu/cgi-bin/hgTrackUi?g=rmsk>
UCSC Genome Browser: <https://genome.ucsc.edu/>

### Bioinformatics datasets

The following datasets associated with human genome assembly hg38 were downloaded from the UCSC Genome Browser (1): human genome sequence, transcription factor binding sites (ENCODE Transcription Factor ChIP-seq), DNase I Hypersensitivity sites (ENCODE Digital DNaseI Hypersensitivity Clusters), ENCODE cis-Regulatory Elements (cCREs) (candidate promoters, candidate proximal enhancers, candidate distal enhancers, CTCF-bound cCREs). We downloaded the annotated list of human protein-coding gene coordinates from ENSEMBL via the biomaRt R package (2,3).

### Analysis of the frequency distribution of pG4-BS occurrences within gene regions

We propose that the molecular microevolution of pG4-BS in a genome could be modeled by the time-continuous Kolmogorov birth and death random process (BDP) (4,5). This is a one-step transition Markov process between neighbor states mediated by sporadic and inducible probabilistic mechanisms. At the stationary regime, the probability distribution function (PDF) is given by Kolmogorov–Waring (K–W) function (4,5). In our case, a state of the process is interpreted as the number of pG4-BSs at a given evolution time $t$, where the birth and death rates are considered as the ‘gain’ and ‘lost’ rates of a single sequence for a given state $m$ counted over genome. We utilized the K–W PDF to characterize and fit the Empirical Frequency Distribution (EFD) of the number of pG4-BS within a given gene (including its flanks (see Methods)) of the human genome. The probabilities of states can be described by the function:

$P\left( X= m;a,b,\theta\right):= p_{m} = p_{0}\frac{B(a,m)}{B(b,m+1)}\theta^{m},$ (1)

where $0<p_{m}<1,$ $m$ is a random variable, ($m = 1, 2, 3, \ldots$ ); $a,b$ and $\theta$ are the unknown non-negative parameters of our PDF.$B(a,m)$and $B(a,m+1)$ are Beta Integral functions. These parameters are defined by the ratios of the transition rates of the BDP. Additionally, we request that the parameter $0<\theta\leq1$*,* which is the sufficient condition for the existence of stationary PDF. Probability $p_{0}=p_{0}(a,b, \theta)$ is the probability of a non-observed event. If $0<p_{0}<1$ then the probabilities can be normalized

$$\sum_{m=0}^{\infty} p_{m}=1$$

Note, if $\theta$ =1, and additionally, $b > \alpha> 0$, the probability of a non-observed event is estimated via the formula $p_{0} =(1 - \frac{\alpha}{b})$ and instead the equation (1) we can use

$p_{m+1}= \theta\frac{(a+m)}{b+m+1}p_{m}$ (2)

More details about the properties of K-W PDF have been described in (4,5).

Note that not all possible states $m$ =0,1,2,… are observed in our data. observed data (EFD) the data set may be incomplete. Due to our pG4-BS model’s assumptions and search algorithm data set may be incomplete. If so, a fraction of the non-observed random variable in state $X=0$ is not counted, however, our model could be estimated. Also, the number states is a finite number $J$; $J$is the largest random variable value defined by finite sample size, this value is given by our search algorithm.

Therefore, for the parameterization of our PDF, we used the double truncated distribution function in the following form:

$p_{m}^{T}(a,b,\theta)= p_{m}/(\sum_{s=1}^{s=J} p_{s})= \frac{p_{m}}{1- p_{0}- P_{J+1}}$ (3)

where$P_{J+1}= \sum_{s>J}^{\infty} p_{s}$.

Equation (3) matches the common situation of parametrization based on an incomplete/limited data set. In these cases, the frequency of the discrete random variable value 0 is not observed and frequency data in states *J* + 1, *J* + 2, … could be overdispersed. The details of our curve-fitting computational algorithm have been previously published (4).

### Gene ontology (GO) analysis

Gene ontology (GO) enrichment analysis was carried out using the database for annotation, visualization and integrated discovery (DAVID) bioinformatics resources (DAVID 2021) (6) and the Enrichr gene-list enrichment tool (7-9). We used DAVID to determine the enrichment of gene functional annotation terms in protein-coding genes with at least one copy of a pG4-BS. We supplied the list of genes as input and the results contain the annotation term and the Fisher’s Exact *P*-value for each term that shows the statistical significance of the enrichment. We used *Benjamini correction* as the default P-value adjustment for results obtained from DAVID. For EnrichR, we utilized the R plugin of the tool. We included the following databases in our analyses: GO_Molecular_Function_2018, GO_Cellular_Component_2018, GO_Biological_Process_2018, WikiPathways_2019_Human, KEGG_2019_Human, Reactome_2016, InterPro_Domains_2019, Panther_2016. In analyses utilizing Enrichr, we filtered our results based on the reported adjusted p-value. This is a rank-based ranking that is calculated as follows: the Fisher exact test is run for many random gene sets to establish a mean rank and standard deviation from the expected rank of each term in the gene-set library. Finally, a z-score is calculated to determine the deviation from the expected rank.

## 2. Supporting Result

### 1.2.1. Gene ontology analysis of genes encompassing pG4-BS

Next, to determine the potential roles of pG4-BS in the regulation of transcription across different functional classes of genes, we performed GO analysis focusing on the subset of pG4-BS(+) genes that contain at least one regulatory pG4-BS region in their promoter region (area 2kb upstream of the TSS). We utilized the Enrichr gene list enrichment analysis tool to perform our analyses (7-9). Regulatory G4-S were selected based on their overlapping with DNase hypersensitive genomic regions and/or transcription factor binding sites (TFBS). First, we separately analyzed the enrichment of terms for the list of genes associated with regulatory pG4-BS and pG4-CS. Our gene lists contained 9,536 and 4,688 gene symbols, respectively. In the case of pG4-BS(+) genes, we observed a statistically significant enrichment (Adjusted P-value < 0.05) in 252 terms from the included databases. These terms, ranged from ‘regulation of transcription from RNA polymerase II promoter’ and ‘Signaling pathways regulating pluripotency of stem cells’, to ‘Pathways in cancer’ and ‘Hippo signaling pathway’. We performed the same analysis using genes encompassing regulatory pG4-CS and identified enrichment in 75 common terms that were enriched in both pG4-CS(+) and pG4-BS(+) gene lists. These terms include transcriptional regulation related terms (e.g., ‘regulation of transcription from RNA polymerase II promoter (GO:0006357)’, ‘positive regulation of transcription, DNA-templated (GO:0045893)’, terms related to signal-transduction (e.g., ‘Hippo signaling pathway’, ‘potassium channel activity (GO:0005267)’), and those related to disease (e.g., ‘Pathways in cancer’). To assess the validity of these findings we repeated our analysis using same-size gene lists. For this, we used three random samples of the pG4-BS gene list with the same size as that of the pG4-CS gene lists (n = 4,688). We observed the enrichment of 31 common GO terms in the three random replicas (Adj. *P*-value < 0.05, in at least one of the replicas). To further elucidate the potential overlap between pG4-BS and pG4-CS functions, we separately carried out GO for genes only containing regulatory pG4-BS (n = 6,135), genes only containing regulatory pG4-CS (n = 1,287), and genes associated with both types of sequences (n = 3,401). In all three gene subsets, we used three random replicas of 1000 gene symbols. Interestingly, we observed the enrichment of nine out of the 31 previously identified common terms, in the random samples of the genes with both regulatory pG4-BS and regulatory pG4-CS. Our results showed no enrichment of common terms in either gene lists containing only pG4-BS(+) or pG4-CS(+) genes. Additionally, the overall enrichment of terms was much weaker in the ‘unique’ subsets, compared to the genes with both types of regulatory G4-S.

We were also interested in whether regulatory pG4-BS that belong to different sequence models (e.g., G2B2, G3B1 or G3B2) could possess different functions. To test this, we performed a GO analysis, using random same-sized subsets of genes encompassing only one type of regulatory pG4-BS. Our analyses revealed only modest term enrichment for all three models. We did not observe the robust enrichment of any terms in these gene lists (data not shown). This suggests that bulged G4s belonging to different sequence models may be involved in similar biological roles, as opposed to unique ones.

## References

1. Kent, W.J., Sugnet, C.W., Furey, T.S., Roskin, K.M., Pringle, T.H., Zahler, A.M. and Haussler, D. (2002) The human genome browser at UCSC. *Genome research*, **12**, 996-1006.

2. Durinck, S., Moreau, Y., Kasprzyk, A., Davis, S., De Moor, B., Brazma, A. and Huber, W. (2005) BioMart and Bioconductor: a powerful link between biological databases and microarray data analysis. *Bioinformatics*, **21**, 3439-3440.

3. Durinck, S., Spellman, P.T., Birney, E. and Huber, W. (2009) Mapping identifiers for the integration of genomic datasets with the R/Bioconductor package biomaRt. *Nat Protoc*, **4**, 1184-1191.

4. Kuznetsov, V.A. (2003) *Family of skewed distributions associated with the gene expression and proteome evolution*. Elsevier North-Holland, Inc.

5. Kuznetsov, V.A. (2003) Hypergeometric Model of Evolution of Conserved Protein Coding Sequences in the Proteomes. *Fluctuation and Noise Letters*, **03**, L295-L324.

6. Sherman, B.T., Hao, M., Qiu, J., Jiao, X., Baseler, M.W., Lane, H.C., Imamichi, T. and Chang, W. (2022) DAVID: a web server for functional enrichment analysis and functional annotation of gene lists (2021 update). *Nucleic acids research*, **50**, W216-221.

7. Chen, E.Y., Tan, C.M., Kou, Y., Duan, Q., Wang, Z., Meirelles, G.V., Clark, N.R. and Ma'ayan, A. (2013) Enrichr: interactive and collaborative HTML5 gene list enrichment analysis tool. *BMC Bioinformatics*, **14**, 128.

8. Kuleshov, M.V., Jones, M.R., Rouillard, A.D., Fernandez, N.F., Duan, Q., Wang, Z., Koplev, S., Jenkins, S.L., Jagodnik, K.M., Lachmann, A. *et al.* (2016) Enrichr: a comprehensive gene set enrichment analysis web server 2016 update. *Nucleic acids research*, **44**, W90-97.

9. Xie, Z., Bailey, A., Kuleshov, M.V., Clarke, D.J.B., Evangelista, J.E., Jenkins, S.L., Lachmann, A., Wojciechowicz, M.L., Kropiwnicki, E., Jagodnik, K.M. *et al.* (2021) Gene Set Knowledge Discovery with Enrichr. *Current Protocols*, **1**, e90.
