# Supplementary material for: Stable bulged G-quadruplexes in the human genome: identification, experimental validation and functionalization
Source: Nucleic Acids Res. 2023 Apr 24;51(9):4148–77. doi: 10.1093/nar/gkad252 (PMC10201450; doi:10.1093/nar/gkad252)
Supplement: gkad252_Supplemental_Files [file gkad252_supplemental_files.zip › Suppl_Figures_PDF_Revised.pdf]

Supplementary figures

I

G3B1 type pG4-BS
Location chr11:70,703,898-70,703,923
SHANK2 intron 14

(a)

GGGCTGGGCAGTCAGGTGGG
GGGCTGGGCAGTCAGGTGGGG
GGGCAGTCAGGTGGGGAGGG
GGGCAGTCAGGTGGGGAGGGG

(b)

5'- GGGCTGGGCAGTCAGGTGGGGAGGGG -3'

II

G3B2 type pG4-BS
Location chr11:126,438,915-126,438,944
KIRREL3 intron 11

(a)

GGGCAGGGCAGTTGTGCAGGG
GGGCAGTTGTGCAGGGAGGG
GTTGTGCAGGGAGGGTGGG
GTTGTGCAGGGAGGGTGGGG

(b)

5'- GGGCAGGGCAGTTGTGCAGGGAGGGTGGGG -3'

III

G2B2 type pG4-BS
Location chr8:127,737,739-127,737,760
MYC intron 1

(a)

GGGGGTAGGAGAGCGGCTAGGG
GGGGTAGGAGAGCGGCTAGGG
GGGTAGGAGAGCGGCTAGGG

(b)

5'- GGGGGTAGGAGAGCGGCTAGGG -3'

Fig. S1: Multiple examples of G4-S sharing G-stems. (**I**): Overlapping pG4-BS belonging to the G3B1 sequence model (**a**), and the merged region (**b**). (**II**): Overlapping pG4-BS belonging to the G3B2 sequence model (**a**), and the merged region (**b**). (**III**): Overlapping pG4-BS belonging to the G2B2 sequence model (**a**), and the merged region (**b**).

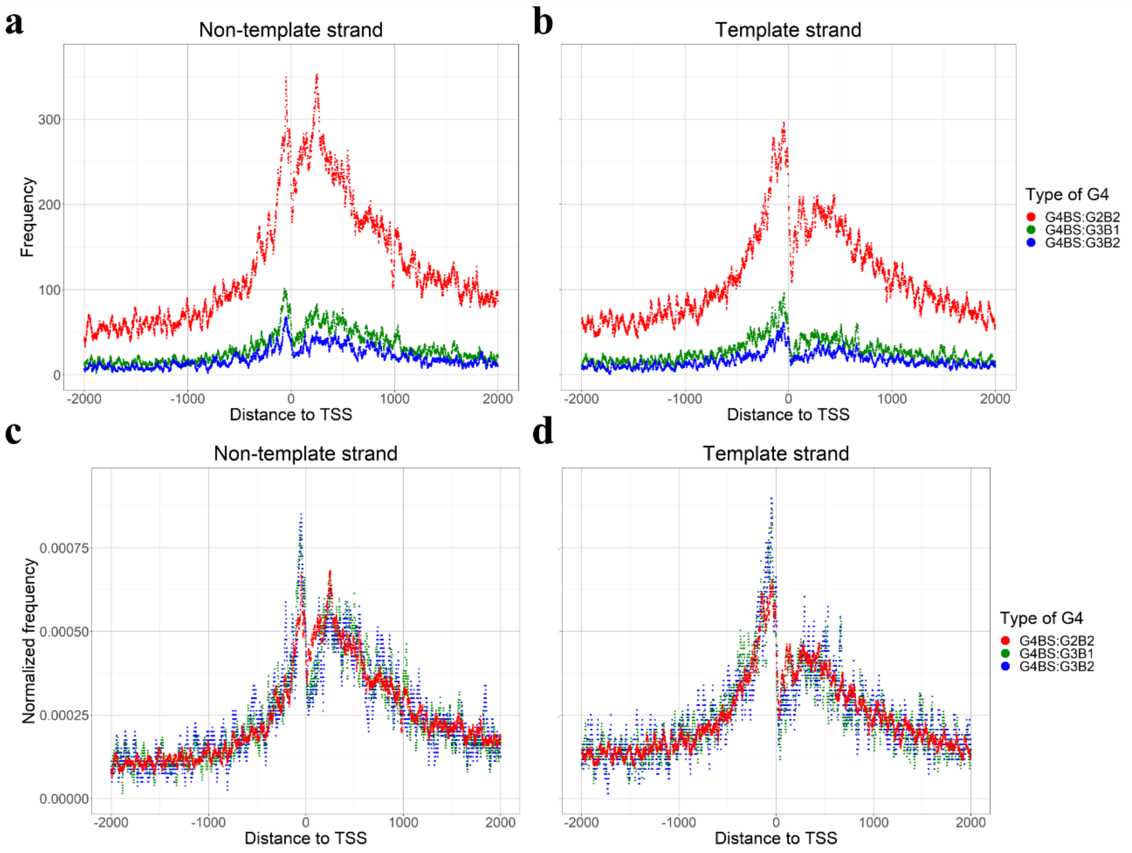

<u>Fig. S2</u>: Sequence model-specific distribution of pG4-BS regions around the TSS of protein-coding genes. (**a-b**) Non-normalized frequency distribution of pG4-BS region counts at each given base position, for the non-template (**a**) and template (**b**) strands. (**c-d**) Normalized frequency distribution functions of pG4-BS region count at each given position for the non-template (**c**) and template (**d**) strands. The single nucleotide counts were normalized to the number of pG4-BS present across all positions.

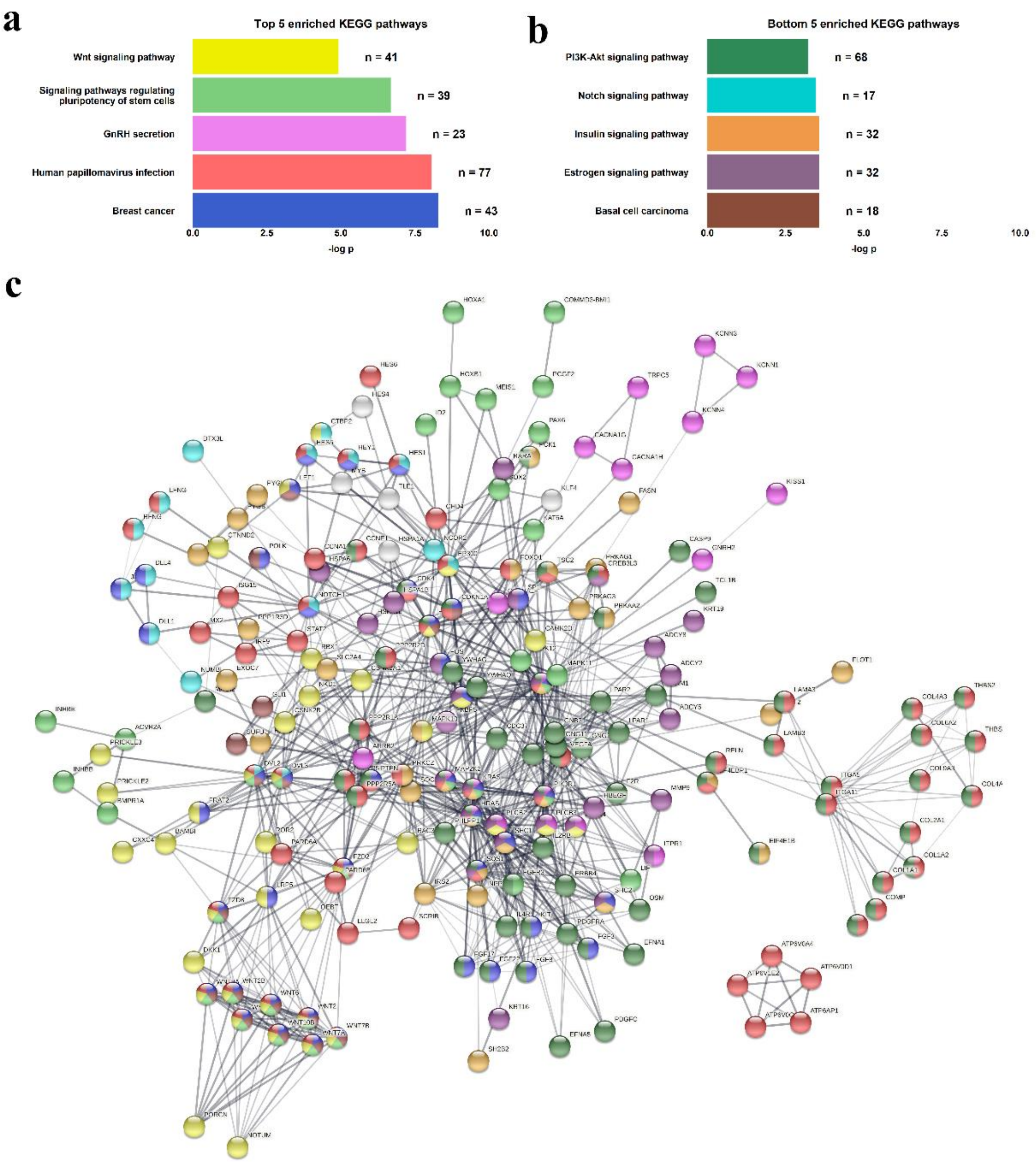

Fig. S3: Plots showing the top 5 (**a**) or bottom 5 (**b**) significantly enriched pathways based on the adjusted *P*-values (cutoff: Adj. *P*-value < 0.05). The values displayed next to the individual bars indicate the number of genes in our gene set that are linked to the given pathway. (**c**) Protein-protein interaction (PPI) network, showing the relationship between the bf-pG4-BS containing genes associated with the top 5 or bottom 5 enriched pathways (n = 217). The colors indicate the following pathways: (red) "Human papillomavirus infection", (blue) "Breast Cancer", (green) "Signaling pathways regulating pluripotency of stem cells",

(yellow) “Wnt signaling pathway”, (pink) “GnRH secretion”, (forest green) “PI3K-Akt signaling pathway”, (teal) “Notch signaling pathway”, (orange) “Insulin signaling pathway”, (purple) “Estrogen signaling pathway”, (brown) “Basal cell carcinoma”.

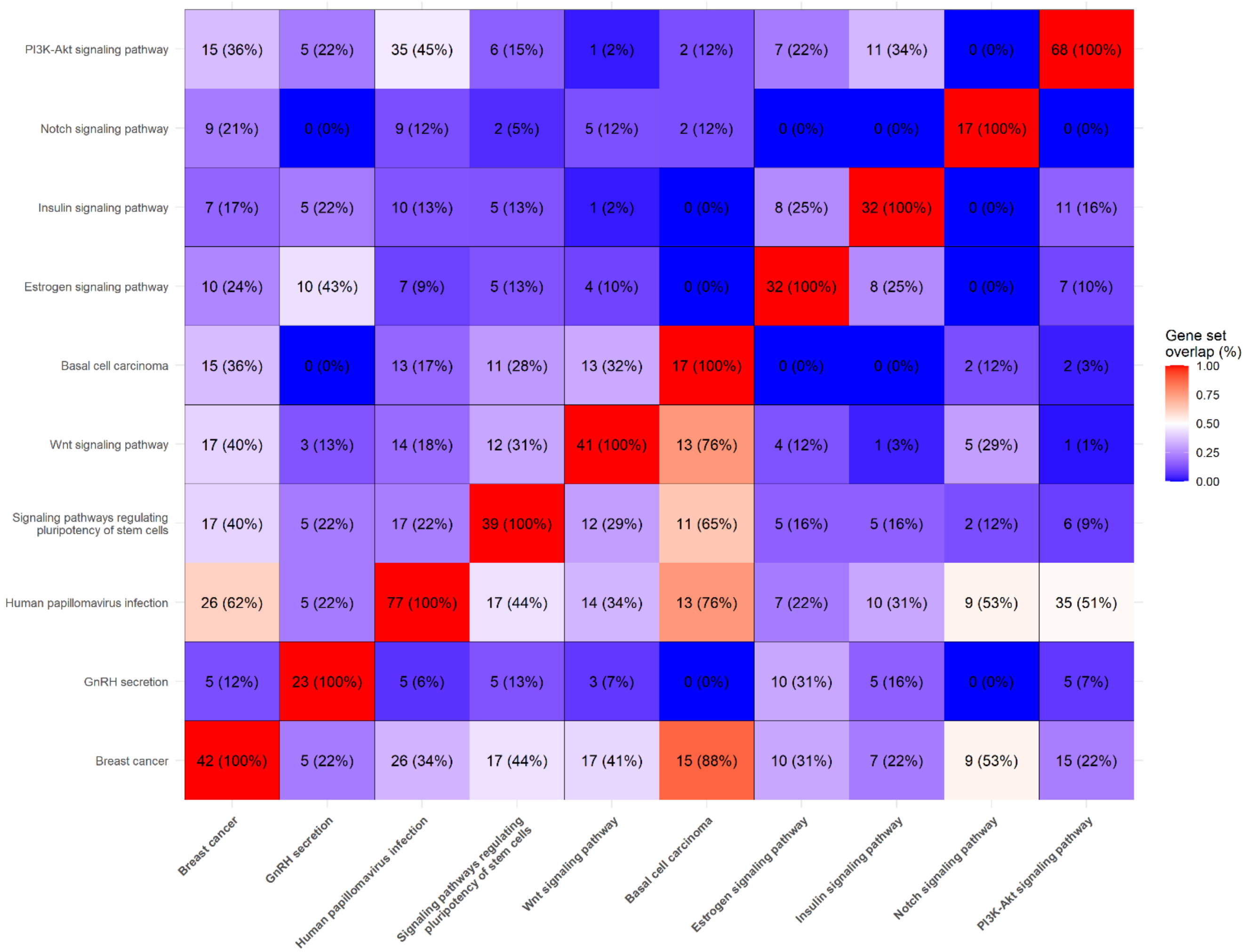

<u>Fig. S4</u>: Heatmap showing the number and ratio of similarity between bf-pG4-BS containing genes associated with each of the selected (top 5 and bottom 5) enriched Kegg pathways. The numbers in each cell indicate the number of genes associated with both pathways at given X and Y-axis positions. The percentages in cells indicate what percentage of the total number of genes associated with the pathways shown on the X-axis is common with the pathways shown on the Y-axis.

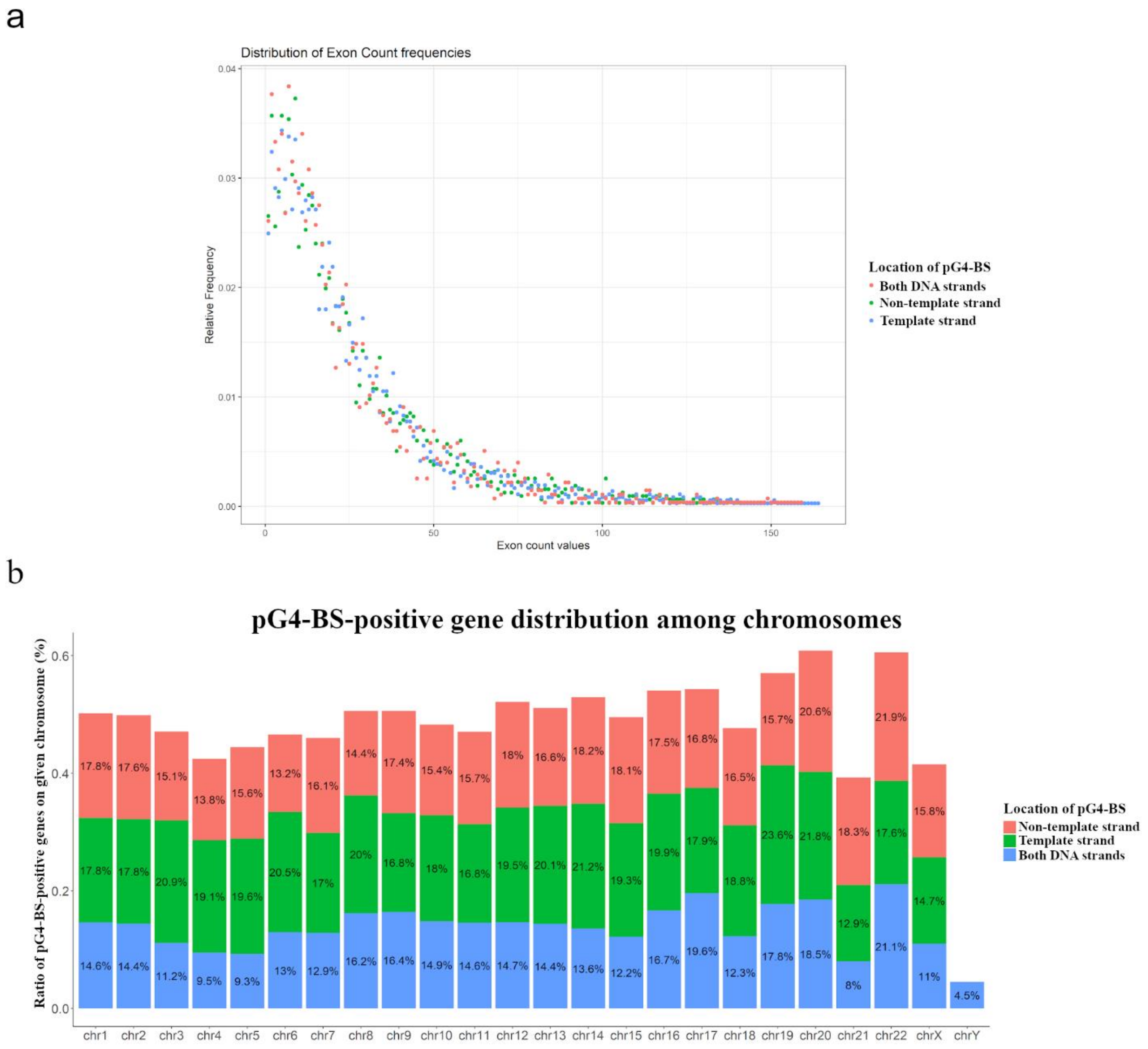

<u>Fig. S5</u>: (**a**): Relative frequency distribution of exon counts in genes containing at least one promoter-associated regulatory pG4-BS region on their Non-template ("NT"), Template ("T") or Both ("BOTH") strands. (**b**): Distribution of genes containing at least one promoter-associated regulatory pG4-BS on their Non-template ("NT"), Template ("T") or Both ("BOTH") strands among the 24 human chromosomes. For both analyses, we normalized the number of genes in each gene set to the total number of human protein-coding genes located on each of the chromosomes.

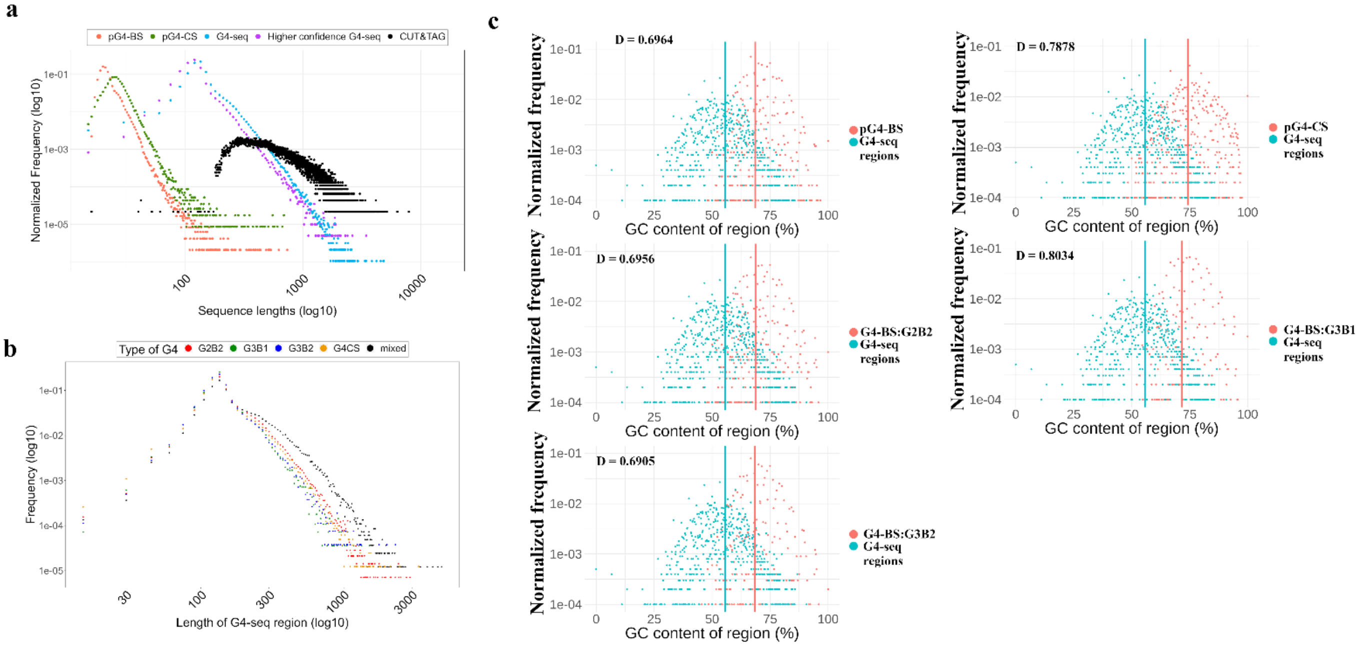

<u>Fig. S6</u>: (**a**): Frequency distribution function of pG4-BS (n = 478,262; mean = 22.79; median = 21; mode = 20), pG4-CS (n = 115,259; mean = 22.7; median = 21; mode = 20), ‘pooled’ G4-seq (n = 949,162; mean = 156.1; median = 135; mode = 135), “higher confidence” G4-seq (n = 200,582; mean = 131.6; median = 120; mode = 120), and ‘pooled’ G4 CUT&Tag (n = 45,968; mean = 708.1; median = 559; mode = 294). We observed skewed, unimodal distributions for all groups. (**b**): Frequency distribution of G4-seq length of regions supported by one or more pG4-S models (n = 358,116). We observed skewed, unimodal distributions for all pG4-BS sequence models. (**c**): Figure showing the frequency distribution functions of GC content of given pG4-S region in percent. We randomly selected 10,000 sequences from each of our datasets and compared the GC% of regions belonging to pG4-BS models, bulk pG4-BS and pG4-CS with those of G4-seq regions. The y-axis is showing the log10 transformed frequency of individual GC% values in each sample. The values in the upper left corner of the plots indicate the distance metric (D) calculated by the K-S test comparing the empirical cumulative distribution functions (ECDF) between each of the G4-S samples and G4-seq regions. Statistical significance was strong for all comparisons (*P*-value $< 2.20E-16$). The vertical lines in each plot indicate the position of the median value for each distribution function.

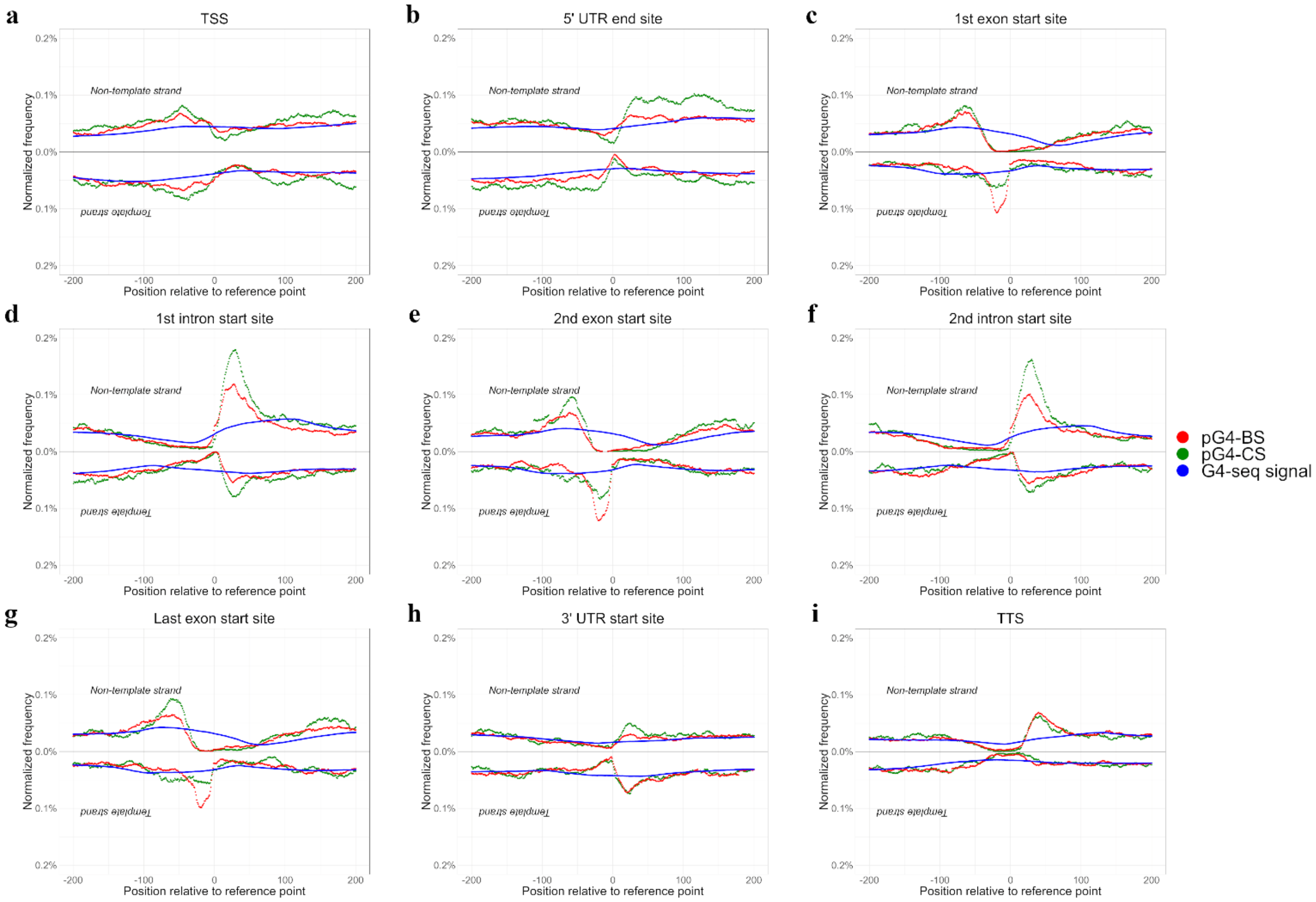

Fig. S7: The normalized experimental frequency distribution function for pG4-BS, pG4-CS and experimentally detected G4 (G4-seq) regions across major gene segments for the non-template and template strands. For this analysis, we studied the following gene segments and their vicinity: (**a**): TSS; (**b**): termination site of 5' UTR; (**c, e, g**): initiation sites of 1st (**c**); 2nd (**e**) and last exons (**g**); (**d, f**): initiation sites of 1st (**d**) and 2nd (**f**) introns; (**h**): initiation site of 3' UTR; and (**i**): the transcription termination site (TTS) of the gene. The frequency of individual signals was counted at each given base position and normalized for the number of signals (e.g., pG4-BS, pG4-CS or G4-seq signal) present across all positions are plotted over a +/- 2kb region centered on the indicated position of each studied gene segment.

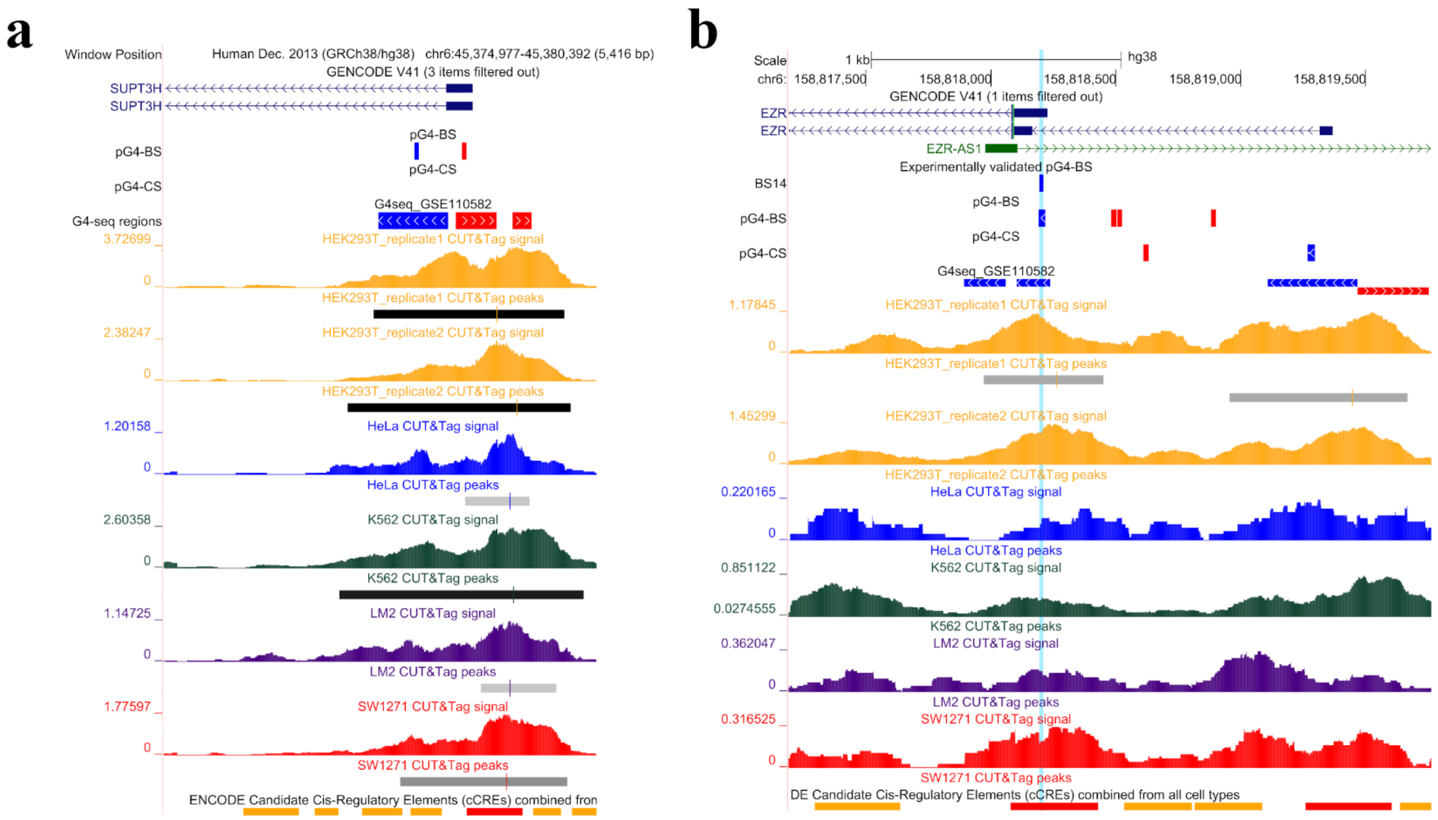

Fig. S8: Genome browser images showcasing common and distinct sites of G4 presence detected via G4 CUT&Tag. (**a**) Example of peaks and signal coverage corresponding to suspected G4-B presence in the promoter region of SUPT3H. (**b**) We observe stronger G4 signal in the approximate region of the EZR promoter, however peak-calling only identified a G4 CUT&Tag peak in one out of the six samples.

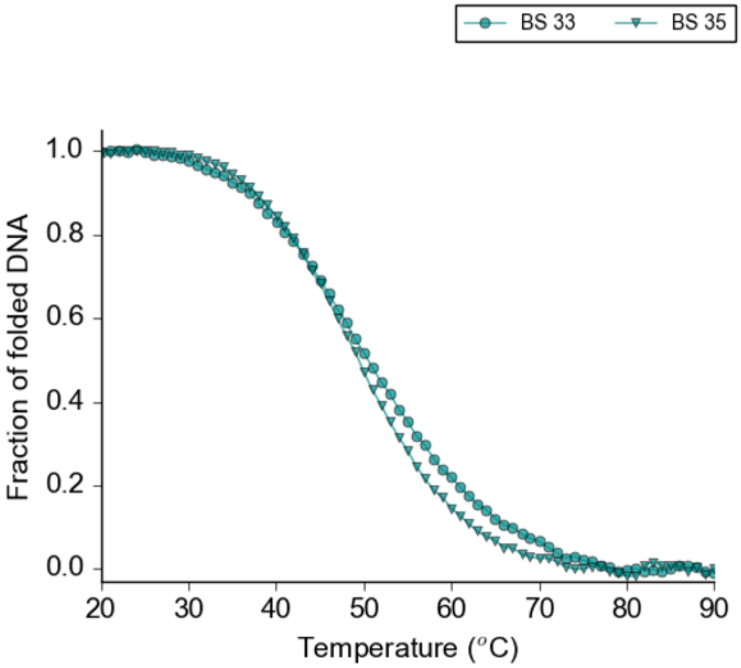

<u>Fig. S9</u>: UV melting curves of selected pG4-BS with two bulges. The results suggest that the G4-B formed by these sequences possess stability comparable to G4s with a single multiple nucleotide bulge.
